# Yinqin Qingfei granules alleviate *Mycoplasma pneumoniae* pneumonia via inhibiting NLRP3 inflammasome-mediated macrophage pyroptosis

**DOI:** 10.3389/fphar.2024.1437475

**Published:** 2024-08-27

**Authors:** Zhe Song, Chengen Han, Guangzhi Luo, Guangyuan Jia, Xiao Wang, Baoqing Zhang

**Affiliations:** ^1^ Department of Pediatrics, Affiliated Hospital of Shandong University of Traditional Chinese Medicine, Jinan, China; ^2^ College of Traditional Chinese Medicine, Shandong University of Traditional Chinese Medicine, Jinan, China

**Keywords:** Yinqin Qingfei granules, *Mycoplasma pneumoniae* pneumonia, macrophage, pyroptosis, NLRP3 inflammasome

## Abstract

**Background:**

*Mycoplasma pneumoniae* pneumonia (MPP) is a prevalent respiratory infectious disease in children. Given the increasing resistance of *M. pneumoniae* (MP) to macrolide antibiotics, the identification of new therapeutic agents is critical. Yinqin Qingfei granules (YQQFG), a Chinese patent medicine formulated specifically for pediatric MPP, lacks a clear explanation of its mechanism.

**Methods:**

The primary components of YQQFG were identified using LC-MS/MS. *In vitro*, RAW264.7 cells infected with MP underwent morphological examination via scanning electron microscopy. Drug-containing serum was prepared, and its intervention concentration was determined using the CCK-8 assay. The active components of YQQFG were molecularly docked with NLRP3 protein using Autodock Vina software. A RAW264.7 cell line overexpressing NLRP3 was created using lentivirus to pinpoint the target of YQQFG. *In vivo*, MPP model mice were established via nasal instillation of MP. Lung damage was assessed by lung index and H&E staining. Pyroptosis-associated protein levels in cells and lung tissue were measured by western blot, while interleukin (IL)-1β and IL-18 levels in cell supernatants and mouse serum were quantified using ELISA. Immunofluorescence double staining of lung tissue sections was conducted to assess the correlation between NLRP3 protein expression and macrophages. The expression of the community-acquired respiratory distress syndrome toxin (CARDS TX) was evaluated by qPCR.

**Results:**

25 effective components with favorable oral bioavailability were identified in YQQFG. Both *in vitro* and *in vivo* studies demonstrated that YQQFG substantially reduced the expression of the NLRP3/Caspase-1/GSDMD pathway, decreasing the release of IL-1β and IL-18, and inhibited MP exotoxin. Molecular docking indicated strong affinity between most YQQFG components and NLRP3 protein. Lentivirus transfection and immunofluorescence double staining confirmed that YQQFG significantly suppressed NLRP3 expression in macrophages, outperforming azithromycin (AZM). The combination of YQQFG and AZM yielded the optimal therapeutic effect for MPP.

**Conclusion:**

YQQFG mitigates inflammatory responses by suppressing NLRP3 inflammasome-mediated macrophage pyroptosis, thereby ameliorating MP-induced acute lung injury. YQQFG serves as an effective adjunct and alternative medication for pediatric MPP treatment.

## 1 Introduction

*Mycoplasma pneumoniae* (MP) is the smallest prokaryote capable of independent survival (Kumar and Kumar, 2023). It spreads through respiratory droplets, leading to periodic outbreaks every 3–7 years. Children are predominantly susceptible (Jacobs et al., 2015). During epidemic years, *M. pneumoniae* pneumonia (MPP) accounts for over 40% of pediatric community-acquired pneumonia (CAP) cases (Kumar, 2018). Currently, macrolide antibiotics, notably azithromycin (AZM), are the first-line treatment for MPP in children (Tsai et al., 2021). However, the extensive use of these antibiotics has led to escalating rates of MP resistance (Kim et al., 2022). Developing new therapeutic agents for MPP is thus critically urgent.

An excessive immune inflammatory response triggered by MP is identified as the primary pathological mechanism of MPP (Deng et al., 2023). Macrophages, the most abundant immune cells in the lungs, are the first to respond to pathogens with phagocytic and pro-inflammatory actions (Byrne et al., 2015). Recent research indicates that MP’s membrane lipoprotein components can activate the NLRP3 inflammasome via the TLRs/NF-κB signaling pathway (Luo et al., 2021). Furthermore, the community-acquired respiratory distress syndrome toxin (CARDS TX) produced by MP possesses ADP-ribosyltransferase (ADPRT) activity, enabling it to catalyze inflammasome assembly directly via NLRP3 protein (Bose et al., 2014). Activation of the NLRP3 inflammasome leads to the conversion of interleukin (IL)-1β and IL-18 precursors into mature forms and the cleavage of the pyroptosis execution protein gasdermin D (GSDMD), which generates N-terminal active fragments (GSDMD-NT) to perforate the cell membrane, resulting in cell death (Yin et al., 2023). The process of pyroptosis not only exacerbates the inflammatory response (Fan and Fan, 2018); it also causes pathogen antigens previously engulfed by macrophages to be released extracellularly, perpetuating infection (Ding et al., 2021). Thus, targeting NLRP3 inflammasome-mediated macrophage pyroptosis offers a promising strategy to mitigate the inflammatory damage associated with MPP.

The use of traditional Chinese medicine (TCM) in treating respiratory tract infections boasts a long-standing history and distinct advantages. Chinese herbal compounds effectively combat pathogens, enhance symptom relief, suppress inflammatory response, and modulate immune function through the strategic combination of various herbs. When used alongside antibiotics, these compounds often yield a synergistic effect (Li et al., 2017; Sun et al., 2020). The primary clinical symptoms of MP infection include fever and cough, often characterized by a prolonged course and the potential to affect multiple organs (Narita, 2010; Wang et al., 2016). This aligns with the TCM concept of “damp-heat evil”, traditionally linked to prolonged inflammatory states marked by intense consumption of body fluids, leading to phlegm and blood stasis accumulation. In TCM theory, dampness, heat, phlegm, and blood stasis are identified as the principal pathological factors in pediatric MPP, with treatments aimed at eliminating dampness and heat, resolving phlegm, and dispelling blood stasis. Yinqin Qingfei granules (YQQFG) are specifically formulated for treating MPP in children, based on these principles. The formulation comprises nine kinds of medicinal materials and has demonstrated effective clinical results. Currently, YQQFG is endorsed as a proprietary blend by the Affiliated Hospital of Shandong University of Traditional Chinese Medicine. However, the mechanism by which YQQFG treats MPP remains to be fully understood. Considering the significant role of NLRP3 inflammasome-mediated macrophage pyroptosis in MP-induced acute lung injury, we postulated that YQQFG possesses the potential to alleviate MPP by inhibiting the activation of the NLRP3 inflammasome and macrophage pyroptosis, and verified this by constructing cellular and murine experimental models in this research.

## 2 Materials and methods

### 2.1 Preparation and component identification of YQQFG

The nine herbs were sourced from the Affiliated Hospital of Shandong University of Traditional Chinese Medicine and verified as authentic by the Pharmacology Laboratory of Traditional Chinese Medicine at Shandong University of Traditional Chinese Medicine. They conformed to the standards outlined in the 2020 edition of the “Pharmacopoeia of the People’s Republic of China”. These herbs were mixed according to the proportions listed in Table 1, subjected to two boiling cycles of 30 min each, and the resulting decoctions were filtered and merged. The mixture was then concentrated under reduced pressure, followed by the addition of dextrin. From each Gram of the original compound preparation, 0.24 g was extracted. The extraction rate of YQQFG was 24%.

**TABLE 1:** Herbal formula of YQQFG.

Chinese name	Latin name	Medicinal part	Weight (g)
Yinchen	*Artemisia scoparia Waldst. et Kit*	dry overground parts	15
Huangqin	*Scutellaria sieversii Bge*	dry roots	9
Huzhang	*Polygonum cuspidatum Sieb. et Zucc*	dry roots and rhizomes	12
Guanghuoxiang	*Pogostemon cablin (Blanco) Benth*	dry overground parts	9
Shichangpu	*Acorus tatarinowii Schott*	dry rhizomes	9
Zhebeimu	*Fritillaria thunbergii Miq*	dry bulbs	9
Houpo	*Magnolia officinalis Rehd. et wils*	dry barks, root barks, and branch barks	9
Taoren	*Prunus davidiana (Carr.) Franch*	dry mature seeds	9
Gancao	*Glycyrrhiza aspera Pall*	dry roots	6

Adding methanol containing 4 ppm of 2-Amino-3-(2-chloro-phenyl)-propionic acid, we prepared samples from six different production batches of YQQFG. Then, we subjected them to vortexing, grinding, ultrasonication, and centrifugation in sequence, and collected the supernatants for testing. LC analysis was carried out with the use of an ACQUITY UPLC^®^ HSS T3 column (2.1 × 100 mm, 1.8 µm) and a Thermo Vanquish UPLC system. We used acetonitrile and ammonium formate (5 mM) for gradient elution in the negative ion mode, and for the positive ion mode, we used 0.1% formic acid in acetonitrile (v/v) and 0.1% formic acid in water (v/v). Thermo Q Exactive mass spectrometers equipped with electrospray ionization were used for MS detection. Concurrent acquisition of MS1 and MS/MS data was carried out in both ion modes. Supplementary Data Sheet S1 details the elution procedure and experimental parameters.

The R XCMS software package was used for feature detection, retention time (RT) correction and alignment. The MS identification of substances was achieved by public spectral databases including HMDB (http://www.hmdb.ca), massbank (http://www.massbank.jp/), LipidMaps (http://www.lipidmaps.org), mzclound (https://www.mzcloud.org), and KEGG (https://www.genome.jp/kegg/). At the same time, the MS/MS data were matched with the fragment ions in the database to achieved the MS/MS identification of the substances. Finally, the TCMSP database (https://old.tcmsp-e.com/tcmsp.php) was employed to screen the identified substances, yielding the active components of YQQFG.

### 2.2 Preparation of drug-containing serum

Forty female 7-week-old Wistar rats, weighing 180–200 g, were acquired from Beijing Weitonglihua Laboratory Animal Co., Ltd. (Beijing, China) and housed in the SPF animal facility at the Experimental Center of Shandong University of Traditional Chinese Medicine. Following a week of acclimatization, the rats were randomly assigned to four groups: drug-free serum, YQQFG serum, AZM serum, and YQQFG + AZM serum, with 10 rats per group. The average weight of rat’s post-acclimatization was 210 g. The dosage of rats was converted according to the dosage of 6-year-old children (standard weight 20 kg). According to product guidelines, the daily oral dosage of YQQFG for 6-year-old children is 12 g, equivalent to 600 mg/kg. Consequently, the dosage for rats was calculated as 6.3 × 600 mg/kg × 0.21 kg ≈ 794 mg. Similarly, the children’s oral dosage of AZM (Pfizer Inc., specification: 100mg/bag, approval number: Z20220088000) is 10 mg/kg, leading to a rat dosage of 6.3 × 10 mg/kg × 0.21 kg ≈ 13.2 mg. The administration regimen was as follows: the YQQFG serum group received 2 mL of YQQFG solution (397 mg/mL) intragastrically daily; the AZM serum group received 2 mL of AZM solution (6.6 mg/mL) daily; the YQQFG + AZM serum group was administered YQQFG by gavage daily, followed by AZM 6 h later; the drug-free serum group received 2 mL of distilled water daily for 7 days. Blood was collected from the abdominal aorta of rats sedated with pentobarbital sodium 2 h after the final gavage. The serum underwent separation, inactivation at 56°C for 30 min, filtration, sterile packaging, and storage at −80°C. The above operations have been approved by the Animal Ethics Committee of Shandong University of Traditional Chinese Medicine (approval number: SDUTCM20220524001).

### 2.3 MP culture and concentration determination

The MP standard strain (ATCC-15531) was sourced from the American Type Culture Collection (Manassas, Virginia, United States). Firstly, MP medium was prepared. In short, 2.04 g *Mycoplasma* broth base (Oxoid, CM1166) was dissolved in 79 mL ultrapure water, and 2 mL 50% glucose injection (Hubei Kelun Pharmaceutical Co., Ltd., approval number: H42021188) and 1 mL 0.5% phenol red sterile solution (Solarbio, G1230) were added. After autoclaving, 50 g of *Mycoplasma* supplement G (Oxoid, SR0059C) was incorporated, thoroughly mixed, and stored at 4°C for future use. The MP strain was revived in the prepared medium and cultured in an incubator at 37°C with 50% humidity. The medium was monitored until the color transitioned from red to yellow, indicating a need for subculturing at a ratio of 1:10.

Due to the minuscule size of MP, conventional turbidimetric methods are unsuitable for quantification. Thus, color change units (CCU) were employed to estimate MP concentration (Calus et al., 2010). The procedure involved labeling dilutions: 10^1^, 10^2^, … 10^12^ on a 96-well plate, adding 180 μL of MP medium to each well. 20 μL of MP bacterial solution was added to the 10^1^ well, followed by serial transfer of 20 μL from 10^1^ to 10^2^ well after thorough mixing, and so forth, achieving gradient dilution. To prevent evaporation, two to three drops of glycerol (Solarbio, G8190) were added to each well. After continuous observation for 1 month, the color change of the medium was recorded. The concentration of MP was calculated according to 1 × 10^x^ CCU/200 μL, which is, 5 × 10^x^ CCU/mL. Here, “x” represents the maximum dilution factor at which the color change occurs (Calus et al., 2010). The MP concentration established in this study was 5 × 10^8^ CCU/mL (Supplementary Data Sheet S2).

### 2.4 Cell culture

Mouse monocyte-macrophage leukemia cells (RAW264.7) were procured from the Cell Resource Center of Peking Union Medical College. The cells were cultured in DMEM High Glucose Medium (BasalMedia, L110KJ) containing 10% fetal bovine serum (FBS) (Gibco, A3161002C) and 1% penicillin-streptomycin solution (BasalMedia, S110JV). Upon reaching 90% confluency, the cells were subcultured at a ratio of 1:3.

### 2.5 Screening for the optimal intervention time of MP

The MP bacterial solution was sub-packed into 2 mL EP tubes, centrifuged at 12,000 rpm for 20 min, and the supernatant was discarded. In each EP tube, 1 mL of cell complete medium was added to resuspend the precipitated bacteria. The cells, in their logarithmic growth phase, were seeded into 6-well plates with a cell density of 5 × 10^5^ cells/mL and a volume of 2 mL. The plates were then incubated in a CO_2_ incubator. The following day, cells were randomly assigned to either a control group or one that underwent MP infection at 2/4/6/12/24-h intervals. For all groups except the control, the medium was replaced with cell culture medium containing MP. Supernatants collected at various intervals post-MP infection were analyzed for IL-1β and tumor necrosis factor-α (TNF-α) levels using ELISA kits (Elabscience, E-EL-M0037 for IL-1β and E-EL-M3063 for TNF-α).

### 2.6 Scanning electron microscope (SEM) observation

Cells were cultured in 24-well plates (cell density: 5 × 10^5^ cells/mL, volume: 0.5 mL). Following 12 h of MP infection, the medium was removed. Each well was then treated with 0.5 mL of electron microscope fixative (Servicebio, G1102) and placed at 4°C for 12 h. The samples underwent fixation, dehydration, drying, and conductive treatment sequentially. SEM was used to observe the morphological alterations in cells post-MP treatment.

### 2.7 Screening the intervention concentration of drug-containing serum by CCK-8 method

Cells in logarithmic growth phase were seeded into 96-well plates (cell density: 2 × 10^5^ cells/mL, volume: 100 μL) and cultured in a CO_2_ incubator. The following day, various concentrations of rat serum were added to the FBS-free cell culture medium. The cells were organized into 18 groups: drug-free serum group (2.5%, 5%, 10%, 20%), YQQFG serum group (2.5%, 5%, 10%, 20%), AZM serum group (2.5%, 5%, 10%, 20%), YQQFG + AZM serum group (2.5%, 5%, 10%, 20%), a positive control group (cells + medium, without rat serum), and a negative control group (medium only). After 12 h of intervention with rat serum, the medium was discarded and 100 μL of CCK-8 solution (Elabscience, E-CK-A362) was introduced to every well. Following this, the 96-well plates were dark-incubated for another 2 h. A microplate reader was used to measure the optical density (OD) of each well at a wavelength of 450 nm. Cell viability = $\frac{\text{dosing}\;\text{group}\;\text{OD}\;-\;\text{negative}\;\text{control}\;\text{group}\;\text{OD}}{\text{positive}\;\text{control}\;\text{group}\;\text{OD}\;-\;\text{negative}\;\text{control}\;\text{group}\;\text{OD}}$ .

### 2.8 Cell grouping and treatment

Cells were plated in 10 × 10 mm culture dishes (cell density: 2 × 10^5^ cells/mL, volume: 10 mL). On the subsequent day, the cells were divided into five groups: Control group (cell culture medium containing drug-free serum), Model group (MP + cell culture medium containing drug-free serum), YQQFG group (MP + cell culture medium containing YQQFG serum), AZM group (MP + cell culture medium containing AZM serum), and YQQFG + AZM group (MP + cell culture medium containing combined serum). The concentration of drug-containing serum in the cell culture medium was determined based on the results from the CCK-8 assay. Following the intervention, cells and cell supernatants from each group were collected.

### 2.9 Molecular docking analysis

Given that NLRP3 protein activation is pivotal in inflammasome assembly and central to the process of pyroptosis (Fu and Wu, 2023), molecular docking was employed to predict the binding ability of YQQFG’s main active components with NLRP3 protein. These chemicals’ molecular structures were obtained from the PubChem database (https://pubchem.ncbi.nlm.nih.gov/). The three-dimensional crystal structure of NLRP3 protein (PDB ID: 8WSM) was obtained from the Protein Data Bank database (https://www.rcsb.org/). Autodock Vina software was used to perform the docking calculations, and Discovery Studio software was used for visualization.

### 2.10 Cell transfection

In this study, the synthesis of the *NLRP3* gene, construction of the plasmid vector and packaging of the lentivirus were entrusted to Hanbio Biotechnology Co., Ltd. (Shanghai, China). The second generation of RAW264.7 cells were seeded into 6-well plates and divided into the NLRP3 overexpression (NLRP3^−OE^) group and the empty vector lentivirus (LV^−Con^) group. Upon reaching 60% cell density, the culture medium was supplemented with the virus solution at an MOI of 20 for transfection. After 72 h of transfection, puromycin (30 μg/mL) (MCE, HY-K1057) was applied to select successfully transfected cells. Transfection efficiency was assessed using quantitative real-time PCR (qPCR).

### 2.11 Animal modeling and administration

Sixty male 5-week-old BALB/c mice, weighing 12–14 g, were acquired from Beijing Weitonglihua Laboratory Animal Co., Ltd., and housed in the biosafety level-2 (BSL-2) animal room. Mice were randomly allocated into five groups: Control, Model, YQQFG, AZM, and YQQFG + AZM, with each group further subdivided into two subgroups based on gavage duration: 3 and 7 days. Following a week of acclimatization, the mice were anesthetized with isoflurane (RWD Life Science Co., Ltd., R510-22-10). The Control group received 100 μL of normal saline intranasally, while the other groups were administered 100 μL of MP bacterial solution (5 × 10^8^ CCU/mL) nasally for three consecutive days. Subsequent gavage of the designated drugs commenced the following day. Dosages for the mice were adapted from those for 6-year-old children (standard weight 20 kg), using a mouse-to-human dose conversion factor of 9.01 (Dou et al., 2024). The calculated doses were 86 mg/d for YQQFG and 1.4 mg/d for AZM for mice averaging 16 g in weight. The gavage regimen was structured as follows: (1) the Control and Model groups received 0.2 mL of distilled water for either 3 or 7 days; (2) the YQQFG group was administered 0.2 mL of YQQFG solution (430 mg/mL) for either 3 or 7 days; (3) the AZM group received 0.2 mL of AZM solution (7 mg/mL) for 3 days, followed by 0.2 mL of distilled water daily for the remaining 4 days; (4) the YQQFG + AZM-3d group received 0.2 mL of YQQFG solution administered via gavage, followed 6 h later by 0.2 mL of AZM solution for 3 days. In the YQQFG + AZM-7d group, for the first 3 days, the mice received the same treatment as the YQQFG + AZM-3d group. However, for the remaining 4 days, the mice were administered 0.2 mL of YQQFG solution daily, followed by 0.2 mL of distilled water 6 h later. The next day after the end of administration, the mice were anesthetized with isoflurane, and blood was collected from the orbital venous plexus. Serum was then separated and stored at −80°C. The left lung was preserved in tissue fixative, and the right lung was cryopreserved. These procedures received approval from the Animal Ethics Committee of Shandong University of Traditional Chinese Medicine (approval number: SDUTCM20220516002).

### 2.12 Calculate the lung index in mice

The body weight of the mice was recorded daily. During dissection, the lung tissue was rinsed with normal saline and then weighed after blotting with filter paper. Lung index = lung weight (mg)/body weight (g).

### 2.13 Histological evaluation

Following a 24-h fixation in tissue fixative, the lung tissue was dehydrated and embedded in paraffin. H&E staining was performed on Sections 5 μm thick. An upright microscope was used to examine the pathological conditions of lung tissue in each group.

### 2.14 qPCR detection

Total RNA was extracted from cells and lung tissues using the SPARKeasy Improved Tissue/Cell RNA Kit (SparkJade, AC0202) and quantified with an ultra-micro spectrophotometer. RNA was reverse transcribed to cDNA using the SPARKscript II RT Plus Kit (With gDNA Eraser) (SparkJade, AG0304). The qPCR reactions were set up according to the manufacturer’s instructions using 2 × SYBR Green qPCR Mix (SparkJade, AH0104) and performed on a Roche LightCycler 480 II instrument. The qPCR primers were synthesized by Sangon Biotech Co., Ltd. (Shanghai, China) (Table 2). The relative expression of target genes was calculated by the 2^−ΔΔCt^ method with GAPDH serving as the internal reference gene.

**TABLE 2:** Primers for qPCR.

Genes	Primers (5′→3′)
*CARDS TX*	F: CAT​GAT​TGC​CAA​CAC​CAG​GAA​TAG​C
	R: AAA​CGA​TAG​CGA​AGC​GGA​AGT​ACC
*NLRP3*	F: GCT​GCG​ATC​AAC​AGG​CGA​GAC
	R: CCA​TCC​ACT​CTT​CTT​CAA​GGC​TGT​C
*GAPDH*	F: GGT​TGT​CTC​CTG​CGA​CTT​CA
	R: TGG​TCC​AGG​GTT​TCT​TAC​TCC

### 2.15 Western blot (WB)

Total protein was extracted from cells and lung tissues using RIPA lysis buffer (Beyotime, P0013B), and protein concentration was determined using a BCA Protein Assay Kit (Beyotime, P0010). Proteins were separated by SDS-PAGE gel electrophoresis and transferred to a PVDF membrane. The membrane was incubated overnight at 4°C with primary antibodies including NLRP3 (abcam, ab270449), ASC (Cohesion, CPA7120), proCaspase-1 (abcam, ab179515), cleaved Caspase-1 (Proteintech, 22915-1-AP), GSDMD (abcam, ab219800), GSDMD-NT (ABclonal, A22523), and GAPDH (Proteintech, HRP-60004). The following day, the membrane was incubated with secondary antibodies at room temperature for 1 h. Detection was performed using ECL chemiluminescence substrate (Millipore, WBULS0100). Band intensities were analyzed using ImageJ software. The relative expression of the target proteins was calculated with GAPDH as the internal reference.

### 2.16 Immunofluorescence detection

Immunofluorescence double staining was performed on lung tissue sections from mice in the 3-day gavage group using the tyramide signal amplification (TSA) method. Paraffin sections underwent sequential dewaxing, hydration, antigen retrieval, and blocking. Sections were first incubated with NLRP3 primary antibody (Servicebio, GB114320) followed by the appropriate fluorescent secondary antibody. Then the TSA staining solution was added dropwise for signal amplification. After a second antigen retrieval, sections were treated with F4/80 primary antibody (Proteintech, 28463-1-AP) and corresponding fluorescent secondary antibody. Nuclei were stained with DAPI (Servicebio, G1012). A fluorescence microscope was used for imaging. The fluorescence intensity of NLRP3 and F4/80, along with their Pearson’s correlation coefficient (PCC), were quantified using ImageJ software.

### 2.17 Detection of the concentrations of pro-inflammatory factors

Mouse IL-1β ELISA Kit (Elabscience, E-EL-M0037) and Mouse IL-18 ELISA Kit (Elabscience, E-EL-M0730) were used to detect the levels of IL-1β and IL-18 in cell supernatants and mouse serum.

### 2.18 Statistical analysis

The GraphPad Prism 9.0 software was used for statistical analysis and graphical representation. Data were presented as mean ± standard deviation. A one-way ANOVA was employed to assess significant differences between the groups, with a significance level set at *P* < 0.05.

## 3 Results

### 3.1 Identification of components in YQQFG

**FIGURE 1:**
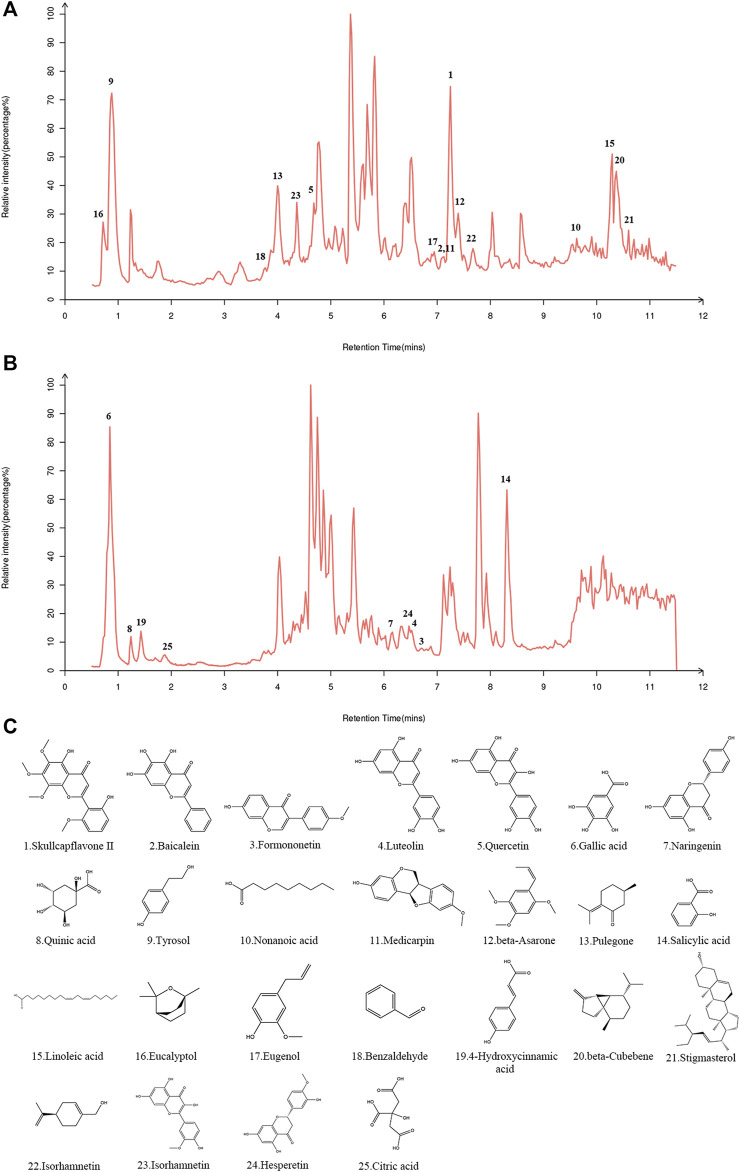
Identification of components in YQQFG. **(A)** Representative TIC in positive ion mode. **(B)** Representative TIC in negative ion mode. **(C)** Molecular structure of components in YQQFG. The molecular structures of the compounds were downloaded from the PubChem database (https://pubchem.ncbi.nlm.nih.gov/) and drawn using the Chem3D software.

In both the positive and negative ion modes, the LC-MS/MS method was used to create the total ion chromatogram (TIC) of YQQFG (Figure 1). Database matching led to the identification of 52 components, comprising 19 flavonoids, 18 organic acids, 6 terpenoids, and 9 other substances (Supplementary Data Sheet S3). Given that oral bioavailability (OB) is a critical pharmacokinetic index reflecting the proportion of an orally administered drug that reaches systemic circulation and exerts pharmacological effects (Xu et al., 2012). Therefore, a criterion of OB ≥ 30% (Zheng et al., 2016; Huo et al., 2022; Ji et al., 2023) was used to select 25 substances in YQQFG with good oral bioavailability from the TCMSP database (Table 3).

**TABLE 3:** 25 components with favorable oral bioavailability in YQQFG.

No.	Components	RT (min)	m/z	Error (ppm)	Precursor type	OB (%)	Source
1	Skullcapflavone II	7.27	375.107	1.5356	[M + H]+	69.51	*Scutellaria sieversii Bge*
2	Baicalein	7.15	271.059	3.9696	[M + H]+	33.52	*Scutellaria sieversii Bge*
3	Formononetin	6.72	267.067	0.3181	[M-H]-	69.67	*Glycyrrhiza aspera Pall*
4	Luteolin	6.53	285.04	2.5400	[M-H]-	36.16	*Polygonum cuspidatum Sieb. et Zucc.;* *Scutellaria sieversii Bge.;* *Prunus davidiana (Carr.) Franch*
5	Quercetin	4.59	303.049	3.8806	[M + H]+	46.43	*Glycyrrhiza aspera Pall.;* *Pogostemon cablin (Blanco) Benth.;* *Polygonum cuspidatum Sieb. et Zucc.;* *Artemisia scoparia Waldst. et Kit.;* *Scutellaria sieversii Bge*
6	Gallic acid	0.83	169.014	2.5087	[M-H]-	31.69	*Polygonum cuspidatum Sieb. et Zucc*
7	Naringenin	6.16	271.061	0.2791	[M-H]-	59.29	*Glycyrrhiza aspera Pall.;* *Magnolia officinalis Rehd. et wils*
8	Quinic acid	1.15	191.056	0.1256	[M-H]-	55.92	*Glycyrrhiza aspera Pall*
9	Tyrosol	0.96	121.066	6.1467	[M + H-H_2_O]+	33.81	*Scutellaria sieversii Bge*
10	Nonanoic acid	9.68	158.153	1.7069	[M]+	40.51	*Scutellaria sieversii Bge.;* *Acorus tatarinowii Schott*
11	Medicarpin	7.15	271.097	0.8263	[M + H]+	49.22	*Glycyrrhiza aspera Pall*
12	beta-Asarone	7.34	209.117	0.5930	[M + H]+	35.61	*Acorus tatarinowii Schott*
13	Pulegone	4.16	153.127	0.0948	[M + H]+	51.60	*Pogostemon cablin (Blanco) Benth.;* *Scutellaria sieversii Bge*
14	Salicylic acid	8.50	137.025	4.9334	[M-H]-	32.13	*Artemisia scoparia Waldst. et Kit*
15	Linoleic acid	10.20	263.236	10.7660	[M + H-H_2_O]+	41.90	*Magnolia officinalis Rehd. et wils.;* *Scutellaria sieversii Bge.;* *Prunus davidiana (Carr.) Franch*
16	Eucalyptol	0.80	154.134	12.3269	[M]+	39.73	*Magnolia officinalis Rehd. et wils.;* *Scutellaria sieversii Bge*
17	Eugenol	7.09	165.091	0.0146	[M + H]+	56.24	*Pogostemon cablin (Blanco) Benth.;* *Magnolia officinalis Rehd. et wils.;* *Scutellaria sieversii Bge.;* *Acorus tatarinowii Schott;* *Artemisia scoparia Waldst. et Kit*
18	Benzaldehyde	3.80	107.049	0.0010	[M + H]+	32.63	*Pogostemon cablin (Blanco) Benth.;* *Acorus tatarinowii Schott*
19	4-Hydroxycinnamic acid	1.30	164.04	1.5158	[M−H]-	43.29	*Scutellaria sieversii Bge.;* *Acorus tatarinowii Schott*
20	beta-Cubebene	10.40	205.196	3.5790	[M + H]+	32.81	*Acorus tatarinowii Schott*
21	Stigmasterol	10.62	395.366	5.5645	[M + H-H_2_O]+	43.83	*Scutellaria sieversii Bge*
22	Perillyl alcohol	7.50	135.117	2.2203	[M + H-H_2_O]+	46.24	*Pogostemon cablin (Blanco) Benth*
23	Isorhamnetin	4.55	317.066	0.0610	[M + H]+	49.60	*Glycyrrhiza aspera Pall.;* *Artemisia scoparia Waldst. et Kit*
24	Hesperetin	6.50	302.075	2.5420	[M]-	70.31	*Magnolia officinalis Rehd. et wils*
25	Citric acid	1.86	191.02	1.9684	[M−H]-	56.22	*Polygonum cuspidatum Sieb. et Zucc.;* *Fritillaria thunbergii Miq*

### 3.2 Screening for the optimal intervention time of MP

**FIGURE 2:**
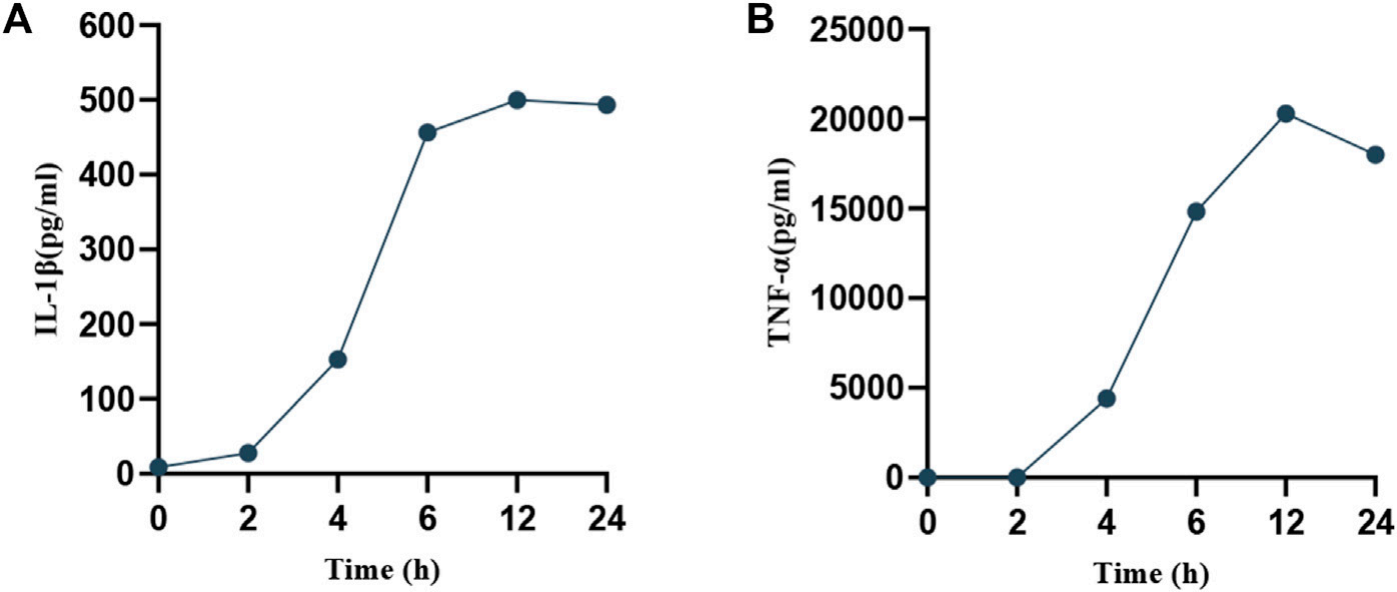
Temporal expression of pro-inflammatory factors in cell supernatants post-MP intervention. **(A)** IL-1β. **(B)** TNF-α.

**FIGURE 3:**
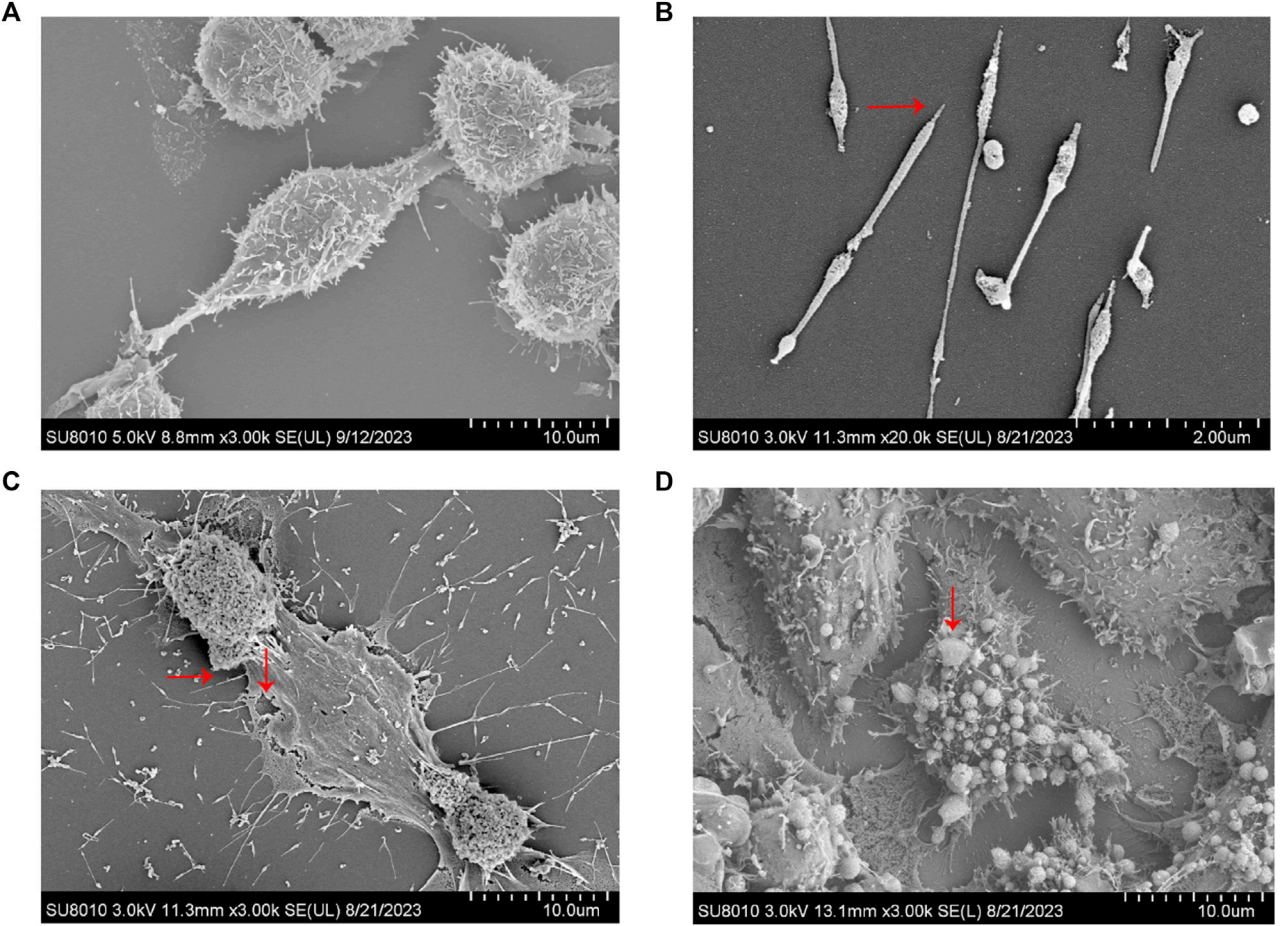
Electron microscopic examination of MP and RAW264.7 cell morphology. **(A)** Normal RAW264.7 cells. **(B)** MP. **(C–D)** Changes in RAW264.7 cells infected with MP for 12 h. →: MP and its attachments; ↓: Cell membrane rupture and pyroptosis bodies.

To ascertain the optimal timing for MP intervention, RAW264.7 cells were infected with MP for periods of 2, 4, 6, 12, and 24 h. Supernatants were collected at each time point to measure the expression of pro-inflammatory factors (IL-1β, TNF-α). As depicted in Figure 2, a rapid increase in the levels of IL-1β and TNF-α was observed starting at 6 h, peaking at 12 h, and subsequently declining. This suggests that the macrophage inflammatory response is most intense at 12 h post-infection, during which pyroptosis likely occurs. To confirm this hypothesis, SEM was utilized to examine changes in cell morphology post-MP infection. Normal RAW264.7 cells appeared round or oval, with a continuous, intact membrane and numerous small pseudopods aiding in phagocytosis (Figure 3A). MP displayed an elongated shape with a attachment organelle at one end (Figures 3B, C). Following 12 h of MP infection, notable changes included increased cell volume, the formation of pyroptotic bodies on the surface, and rupturing of the cell membrane to form pores (Figures 3C, D). These alterations align with the classic morphological features of pyroptosis, indicating that a 12 h MP infection can indeed induce substantial production of pro-inflammatory factors and pyroptosis. Consequently, 12 h was selected as the intervention point for MP in subsequent experiments.

### 3.3 Serum containing YQQFG shows promise in reducing the release of pro-inflammatory factors, inhibiting MP-induced NLRP3 inflammasome activation and pyroptosis

**FIGURE 4:**
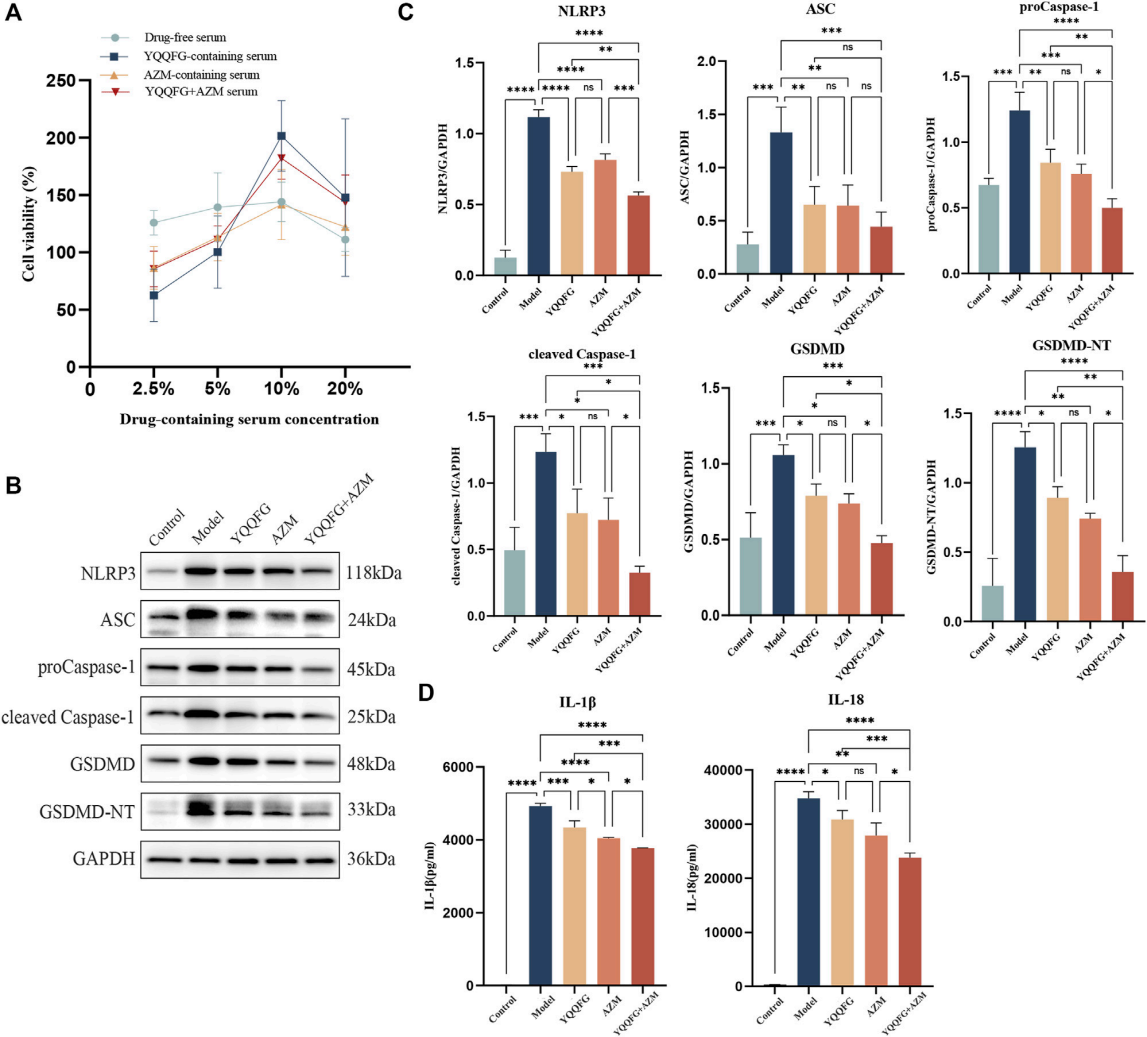
The effect of YQQFG-containing serum on the expression of the NLRP3/Caspase-1/GSDMD pyroptosis pathway induced by MP. **(A)** Effect of different concentrations of drug-containing serum on cell viability. **(B)** Representative WB bands of each protein. **(C)** Statistics on the gray values for each protein. **(D)** Statistics on the expression levels of IL-1β and IL-18 in cell supernatants. Values are the mean ± SD (*n* = 3). ns *P* > 0.05, * *P* < 0.05, ** *P* < 0.01, *** *P* < 0.001, **** *P* < 0.0001.

We began by using the CCK-8 assay to determine the effect of various concentrations of drug-containing serum on cell survival. Figure 4A shows that the medium containing 10% drug-carrying serum had the highest survival rate, thus this was the concentration chosen for subsequent *in vitro* drug intervention treatments. See the WB results in Figures 4B, C. In the Model group, the expression levels of the NLRP3/Caspase-1/GSDMD pathway were significantly elevated (*P* < 0.001), and there was a marked increase in the concentrations of IL-1β and IL-18 in the cell supernatants (*P* < 0.001) (Figure 4D). Serum containing YQQFG, serum containing AZM, and combination serum were all able to successfully reverse these alterations. Protein expression levels along the NLRP3/Caspase-1/GSDMD pathway did not differ significantly between the YQQFG and AZM groups. Crucially, when contrasted with the YQQFG and AZM groups, the YQQFG + AZM group exhibited significantly decreased levels of NLRP3, proCaspase-1, cleaved Caspase-1, GSDMD, and GSDMD-NT protein expression in the cells, as well as a reduction in the content of pro-inflammatory components in the cell supernatants (*P* < 0.05). This suggests that the combined use of YQQFG and AZM can have a synergistic effect.

### 3.4 Molecular docking analysis

**FIGURE 5:**
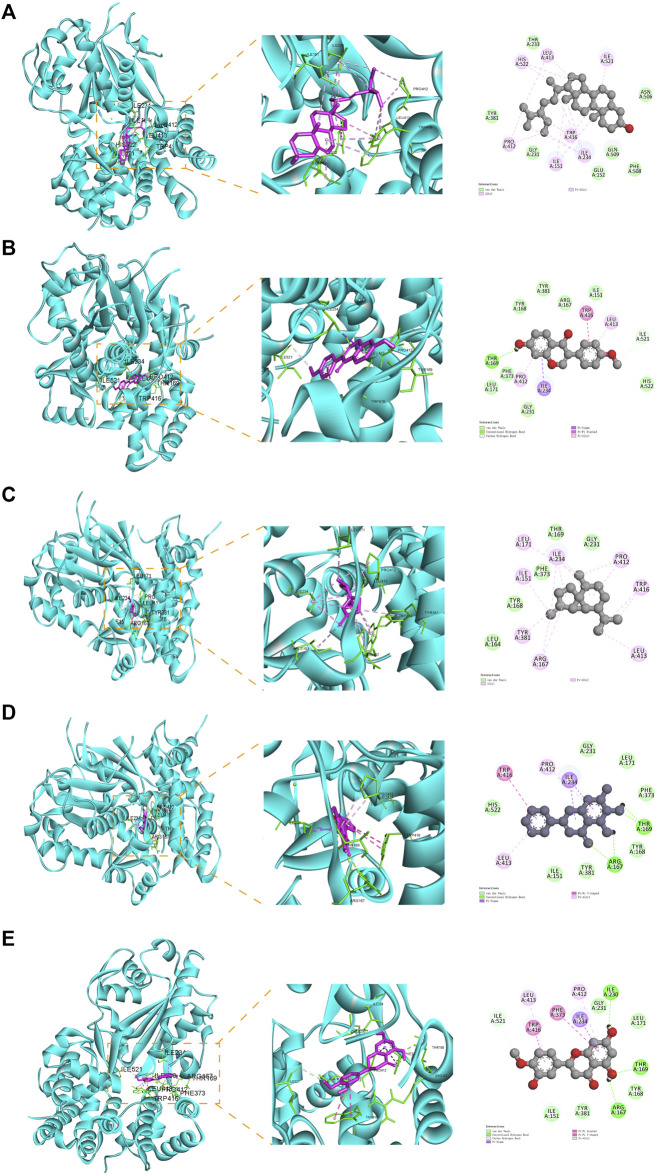
Molecular docking pattern diagram (the five compounds with the strongest affinity to NLRP3). **(A)** Stigmasterol and NLRP3 (binding energy: -8.59). **(B)** Formononetin and NLRP3 (binding energy: -6.99). **(C)** beta-Cubebene and NLRP3 (binding energy: -6.96). **(D)** Baicalein and NLRP3 (binding energy: -6.53). **(E)** Hesperetin and NLRP3 (binding energy: -6.34).

To elucidate the potential of YQQFG in regulating NLRP3, we performed molecular docking of 25 active components from YQQFG with the NLRP3 protein. Typically, if the binding energy of a compound to a target protein is less than −4.25 kcal mol^-1^, this indicates some binding activity and the ability to form a stable complex. A binding energy less than −5.0 kal·mol^-1^ indicates strong binding activity (Ji et al., 2023). As detailed in Table 4, the binding energies of most YQQFG components to NLRP3 were below −4.25 kal·mol^-1^, with 17 components displaying binding energies below −5.0 kal·mol^-1^. To illustrate this interaction, the five compounds with the lowest binding energies were selected for detailed visualization of their binding modes with NLRP3 (Figure 5).

**TABLE 4:** Binding energy of YQQFG active components to NLRP3.

No.	Components	Binding energy (kal·mol^−1^)	No.	Components	Binding energy (kal·mol^−1^)
1	Stigmasterol	−8.59	14	Quercetin	−5.18
2	Formononetin	−6.99	15	Gallic acid	−5.07
3	beta-Cubebene	−6.96	16	Isorhamnetin	−5.04
4	Baicalein	−6.53	17	Perillyl alcohol	−5.03
5	Hesperetin	−6.34	18	Eucalyptol	−4.98
6	Citric acid	−6.18	19	Salicylic acid	−4.97
7	Naringenin	−6.09	20	Nonanoic acid	−4.50
8	4-Hydroxycinnamic acid	−5.85	21	Benzaldehyde	−4.43
9	Pulegone	−5.81	22	Linoleic acid	−4.42
10	Skullcapflavone II	−5.72	23	Eugenol	−4.35
11	beta-Asarone	−5.64	24	Tyrosol	−4.22
12	Luteolin	−5.58	25	Quinic acid	−3.79
13	Medicarpin	−5.53			

### 3.5 NLRP3 overexpression exacerbates MP-induced pyroptosis and inflammatory responses, whereas YQQFG potently inhibits the expression of NLRP3 protein to exert therapeutic effects

**FIGURE 6:**
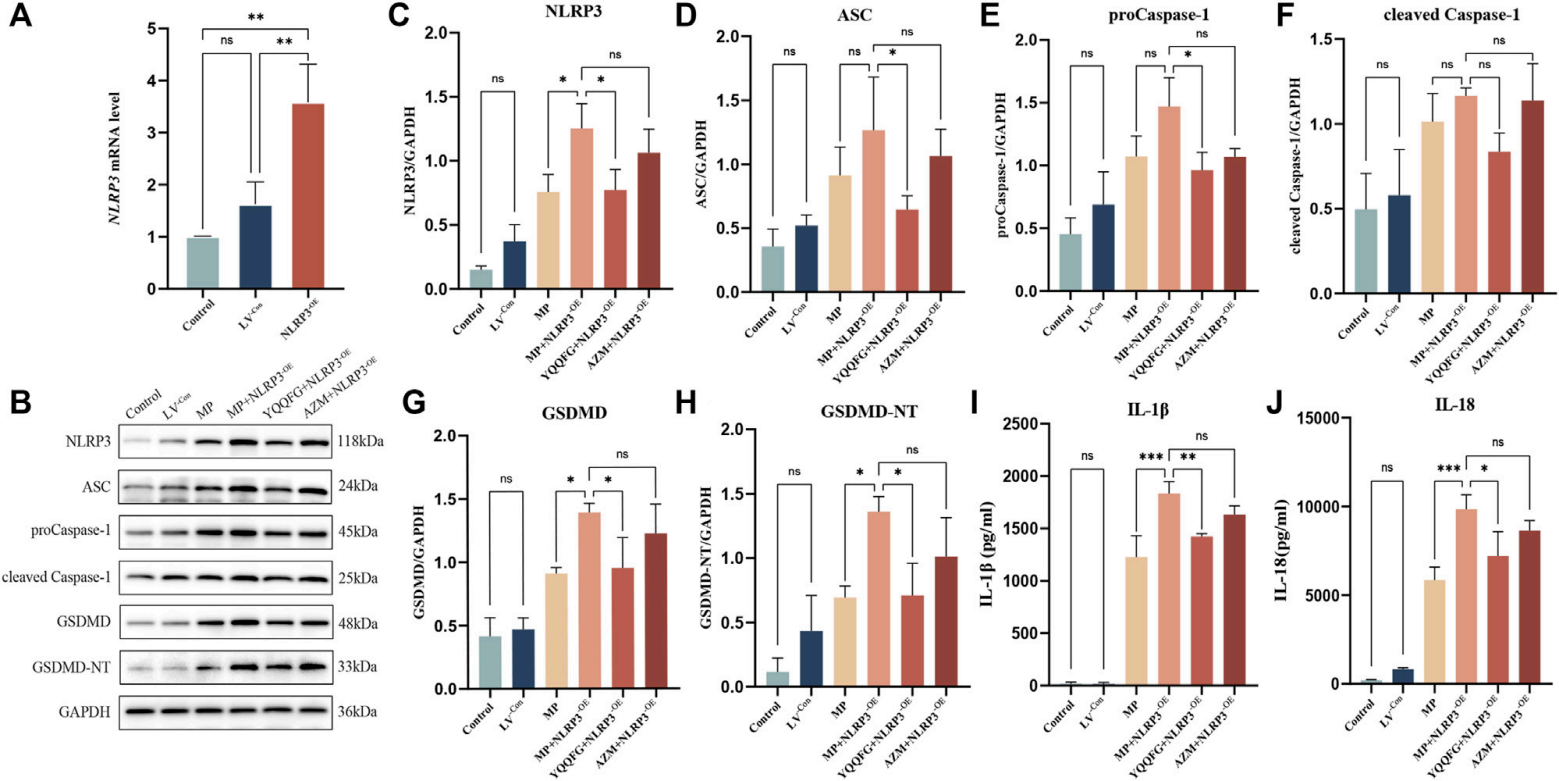
Effects of NLRP3 overexpression on the expression levels of NLRP3/Caspase-1/GSDMD pathway across different cell groups. **(A)** qPCR verification of transfection efficiency. **(B)** Representative WB bands for each protein. **(C–H)** Statistics on the gray values for each protein. **(I–J)** Statistics on the expression levels of IL-1β and IL-18 in cell supernatants. Values are the mean ± SD (*n* = 3). ns *P* > 0.05, * *P* < 0.05, ** *P* < 0.01, *** *P* < 0.001, **** *P* < 0.0001.

Subsequently, we engineered RAW264.7 cells to overexpress NLRP3 via lentiviral transfection (referred to as NLRP3^−OE^). qPCR results demonstrated that NLRP3 expression in NLRP3^−OE^ cells was over threefold higher than in wild-type RAW264.7 cells, confirming successful establishment of a stable NLRP3-overexpressing cell line (Figure 6A). We then examined the impact of YQQFG-containing and AZM-containing sera on the NLRP3/Caspase-1/GSDMD pathway and the levels of IL-1β and IL-18 under conditions of NLRP3 overexpression. WB and ELISA analyses indicated no statistically significant differences in the levels of NLRP3, ASC, proCaspase-1, cleaved Caspase-1, GSDMD, GSDMD-NT, IL-1β, and IL-18 between the LV^−Con^ group and the Control group (*p >* 0.05), suggesting that the vector virus did not alter the pathway’s expression or increase the release of pro-inflammatory factors (Figures 6B–J). However, levels of NLRP3, GSDMD, and GSDMD-NT, as well as the concentrations of IL-1β and IL-18 in the cell supernatants, were significantly elevated in the MP + NLRP3^−OE^ group compared to the MP group (*P* < 0.05), signifying that NLRP3 overexpression exacerbated MP-induced pyroptosis and inflammatory responses. Furthermore, we observed that YQQFG-containing serum still inhibited the activation of the NLRP3/Caspase-1/GSDMD pathway and the release of pro-inflammatory factors following NLRP3 overexpression, but the effect of AZM-containing serum was notably reversed. These findings confirm that YQQFG can specifically modulate the expression of NLRP3 protein, thereby inhibiting inflammasome activation and pyroptosis, and ultimately mitigating MP-induced inflammatory responses.

### 3.6 YQQFG enhances weight recovery in MPP mice, reduces lung index, and alleviates acute lung injury

**FIGURE 7:**
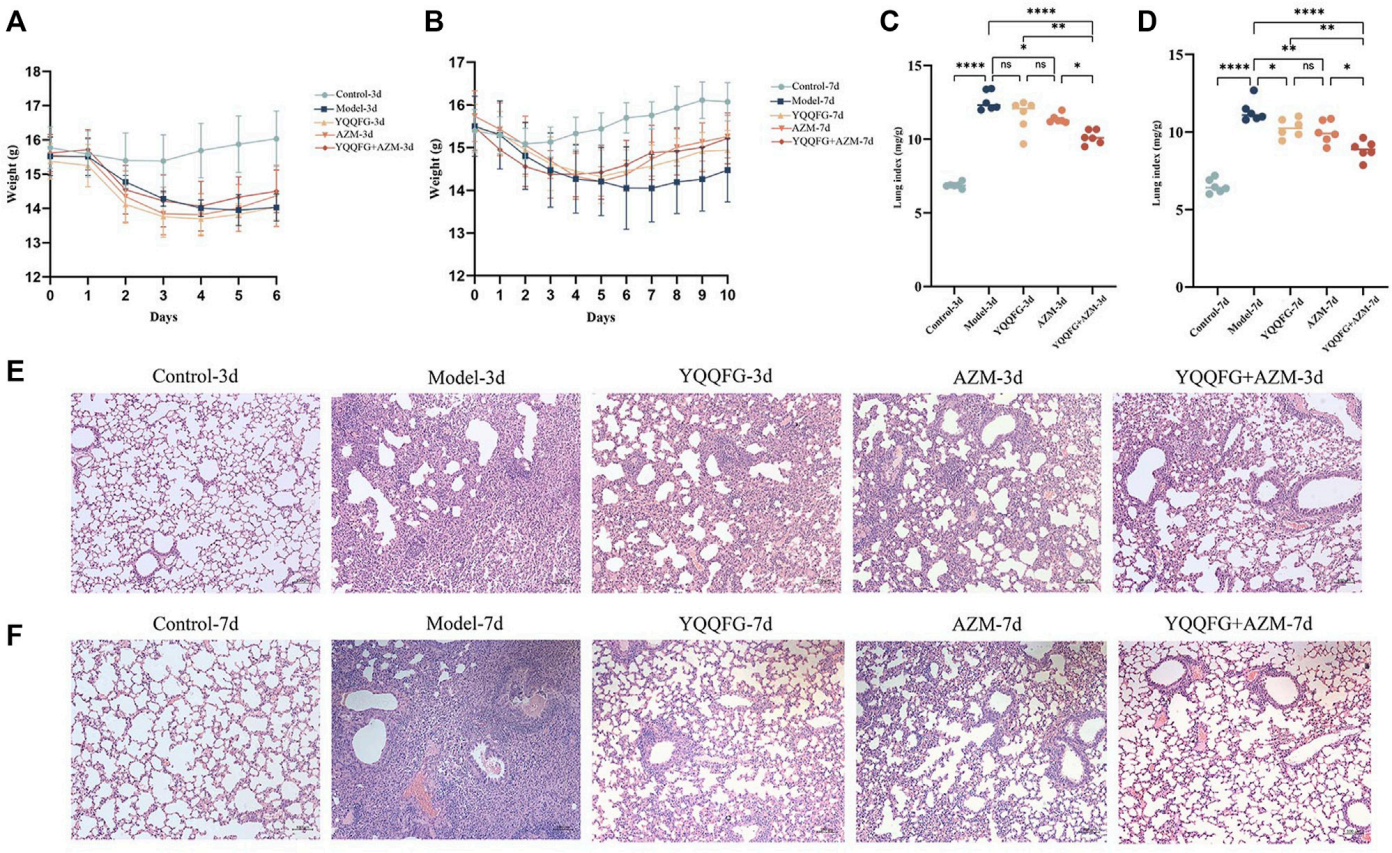
Effect of YQQFG on body weight, lung index, and lung pathology in MPP model mice. **(A)** Body weight change line chart for the 3-day gavage group. **(B)** Body weight change line chart for the 7-day gavage group. **(C)** Lung index for the 3-day gavage group. **(D)** Lung index for the 7-day gavage group. **(E–F)** H&E staining of lung tissue sections for the 3-day and 7-day gavage groups, respectively (Scale: 100 μm). Values are the mean ± SD (*n* = 6). ns *P* > 0.05, * *P* < 0.05, ** *P* < 0.01, *** *P* < 0.001, **** *P* < 0.0001.

Figures 7A, B displays the daily weight changes of mice in each group. The body weight of mice in the Control group slightly decreased on days 2–3 post-modeling but then gradually increased, indicating that inhalation of isoflurane and saline nasal drops did not have a long-term impact on their health. The weight of mice exposed to MP nasal drip decreased significantly. Drug-treated mice began to gain weight on the 5th day (i.e., the second day of gavage). The weight recovery rate of the YQQFG + AZM group was marginally better than that of the YQQFG group and the AZM group. The weight of the Model group exhibited a slight upward trend on the 8th day. The lung index, a common metric for evaluating pathological changes in lung tissue in animal models, showed a significant increase in the Model group (*P* < 0.0001) (Figures 7C, D). YQQFG administration for 3 days had no significant impact on the lung index, but the lung index of the YQQFG + AZM group was significantly lower than that of the AZM group (*P* < 0.05). After 7 days of gavage, compared to the Model group, the lung index in the three drug treatment groups decreased, with no statistical differences between the YQQFG and AZM groups. Figures 7E, F shows H&E staining results indicating that the lung tissue structure of the Control group was clear and intact, with consistent alveolar wall thickness and no hemorrhage, edema, or exudation in the alveoli, nor any inflammatory cell infiltration around the trachea. The lungs of mice in the Model group exhibited severe inflammatory changes, characterized by alveolar loss, lung consolidation, and extensive inflammatory cell infiltration around the alveoli and trachea. As the disease progressed, the extent of inflammatory cell infiltration in the Model-7d group was significantly greater than in the Model-3d group, with some airways completely blocked. The lung tissue of the YQQFG-3d group, the AZM-3d group, and the YQQFG + AZM-3d group showed limited consolidation, disappearance of some alveolar regions, and lower levels of inflammatory cell infiltration compared to the Model-3d group. The YQQFG + AZM-3d group exhibited the best lung ventilation. Longitudinal comparisons across the treatment durations revealed that the lung condition in the 7-day administration groups was significantly improved compared to the 3-day groups. Notably, the lung tissue of mice in the YQQFG + AZM-7d group returned to normal, reaffirming the synergistic effect of combining YQQFG and AZM.

### 3.7 *In vivo* experiments indicate that YQQFG mitigates MPP by suppressing NLRP3 inflammasome activation and macrophage pyroptosis

**FIGURE 8:**
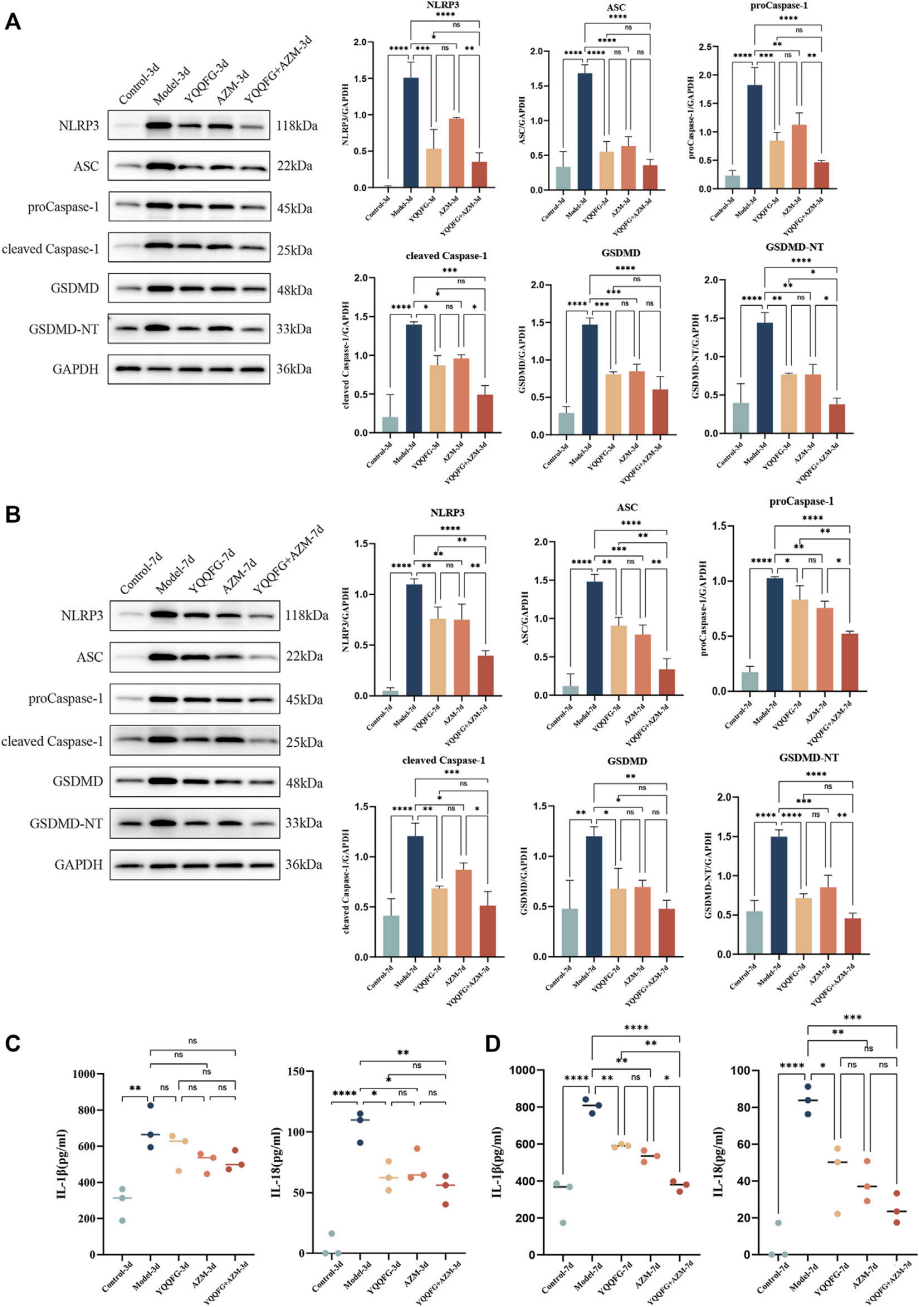
Effects of YQQFG on the NLRP3/Caspase-1/GSDMD pathway in lung tissue and pro-inflammatory factors expression in peripheral serum of mice. **(A, B)** Representative WB bands and statistics on the gray values for proteins in the NLRP3/Caspase-1/GSDMD pathway from the 3-day gavage group and the 7-day gavage group of mice. **(C)** Statistics on the expression levels of IL-1β and IL-18 in peripheral serum of mice in the 3-day gavage group. **(D)** Statistics on the expression levels of IL-1β and IL-18 in peripheral serum of mice in the 7-day gavage group. Values are the mean ± SD (*n* = 3). ns *P* > 0.05, * *P* < 0.05, ** *P* < 0.01, *** *P* < 0.001, **** *P* < 0.0001.

Compared to the Control-3d and Control-7d groups, the NLRP3/Caspase-1/GSDMD pathway proteins in the lung tissues of the Model-3d and Model-7d groups were significantly upregulated (Figures 8A, B), and the levels of IL-1β and IL-18 in peripheral serum increased significantly (*P* < 0.01) (Figures 8C, D). This suggests that the NLRP3 inflammasome-mediated pyroptosis pathway was activated during the acute phase of MPP, enhancing the release of IL-1β and IL-18. Administration of YQQFG or AZM for 3 days effectively reduced the expression of the NLRP3/Caspase-1/GSDMD pathway and the content of IL-18. However, a reduction in IL-1β required 7 days of treatment. Aligning with *in vitro* findings, the results indicate that both YQQFG and AZM are equally effective, but their combination provides the most substantial therapeutic benefit.

**FIGURE 9:**
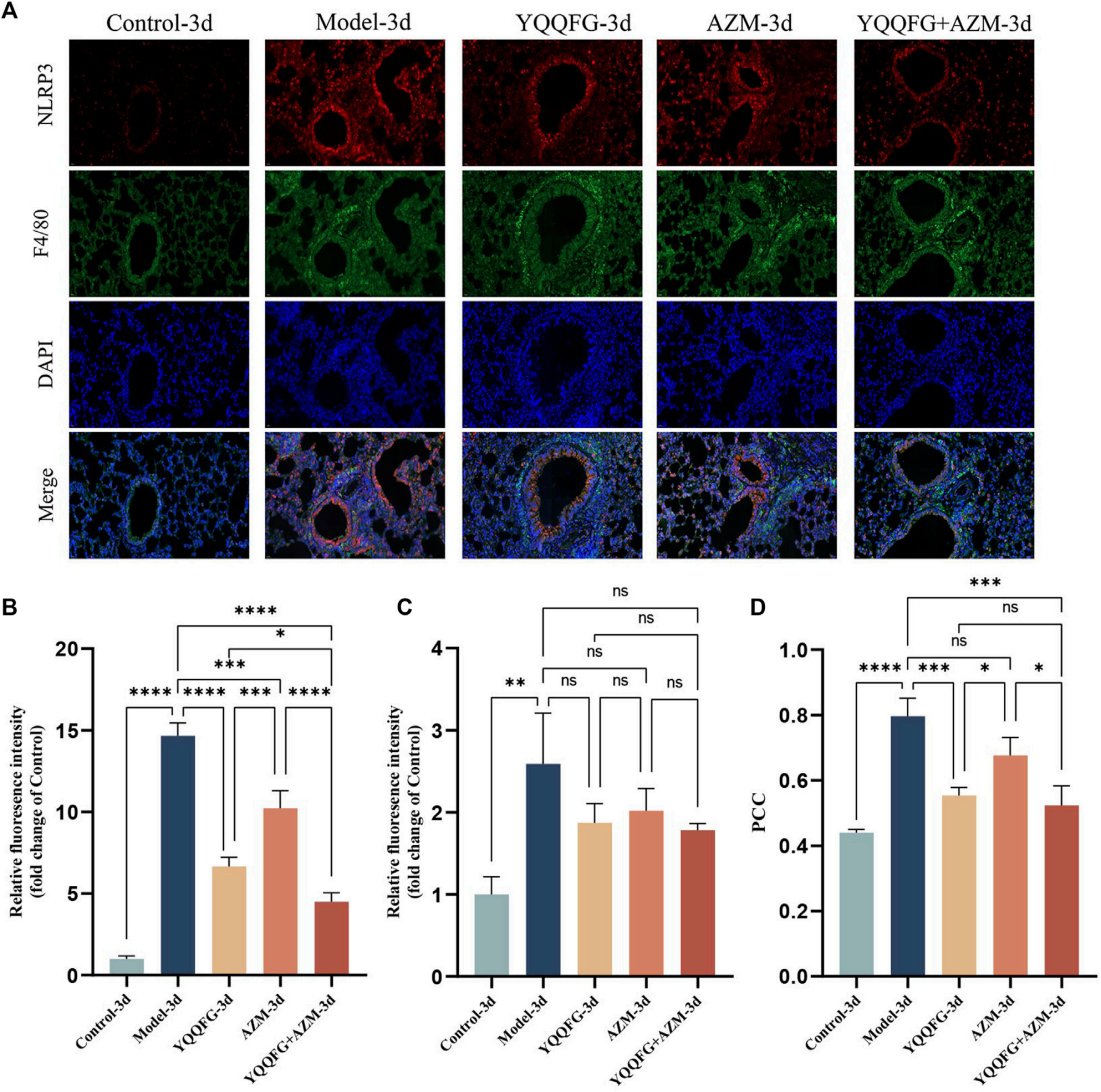
YQQFG can significantly inhibit the expression of NLRP3 protein in macrophages in the lungs of MPP model mice. **(A)** Representative immunofluorescence staining of the 3-day gavage group: red fluorescence indicates NLRP3, green represents F4/80, and blue denotes DAPI (Scale: 20 μm). **(B)** Statistics on relative fluorescence intensity for NLRP3. **(C)** Statistics on relative fluorescence intensity for F4/80. **(D)** Co-localization analysis of NLRP3 and F4/80. Values are the mean ± SD (*n* = 3). ns *P* > 0.05, * *P* < 0.05, ** *P* < 0.01, *** *P* < 0.001, **** *P* < 0.0001.

To explore the relationship between NLRP3 protein expression in lung tissue and macrophages, we conducted immunofluorescence double staining of NLRP3 and mouse macrophage-specific markers (F4/80) on lung tissue sections from the 3-day gavage group. The fluorescence intensity of NLRP3 and F4/80 was significantly enhanced in the Model-3d group (*P* < 0.0001) (Figures 9A–C), indicating NLRP3 protein activation and macrophage infiltration in the lungs of MPP mice. In contrast, the fluorescence intensity of NLRP3 in the YQQFG-3d group, the AZM-3d group, and the YQQFG + AZM-3d group was significantly reduced. PCC, a metric utilized to quantify the extent of protein co-localization, spans a range from −1 to 1, where values approaching 1 signify a stronger degree of co-localization (Dunn et al., 2011). As demonstrated in Figure 9D, the PCC values in the Model-3d group were significantly elevated (*P* < 0.0001). In comparison to the Model-3d group, the PCC values in both the YQQFG-3d and YQQFG + AZM-3d groups exhibited significant decreases (*P* < 0.001), whereas no statistical difference was observed between the AZM-3d and Model-3d groups. This suggests that MP predominantly activates NLRP3 protein in macrophages, leading to inflammasome assembly and macrophage pyroptosis, thereby causing acute lung injury. YQQFG effectively inhibits NLRP3 protein activation in macrophages, demonstrating a significant advantage over AZM in this regard.

### 3.8 YQQFG moderately reduces the release of MP exotoxin

**FIGURE 10:**
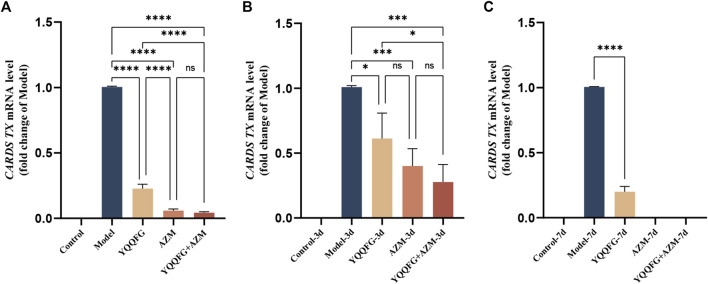
YQQFG can inhibit the release of MP exotoxin to a certain extent. **(A)** Comparison of CARDS TX expression in cells of each group. **(B)** Comparison of CARDS TX expression in lung tissue of mice in the 3-day gavage group. **(C)** Comparison of CARDS TX expression in lung tissue of mice in the 7-day gavage group. Values are the mean ± SD (*n* = 3). ns *P* > 0.05, * *P* < 0.05, ** *P* < 0.01, *** *P* < 0.001, **** *P* < 0.0001.

CARDS TX is currently the only identified MP exotoxin (Li et al., 2019). Studies have shown that after endocytosis into cells, CARDS TX transfers the ADP ribose groups to specific amino acids of NLRP3, thereby directly initiating the assembly of the NLRP3 inflammasome (Bose et al., 2014). Consequently, we employed qPCR to assess the relative expression of CARDS TX mRNA in MP-infected RAW264.7 cells and MPP model mice (Figure 10). In the *in vitro* model, CARDS TX expression was not detected in the Control group. Compared with the Model group, the expression of CARDS TX in the drug intervention groups was significantly reduced (*P* < 0.0001). However, the inhibitory effect of YQQFG on CARDS TX was less pronounced than that of AZM. In the *in vivo* model, similar results were observed. The expression of CARDS TX in the lung tissue of mice decreased following 3 days of treatment with YQQFG, AZM, or their combination (*P* < 0.05). After 7 days of treatment, CARDS TX was undetectable in the AZM-7d group and the YQQFG + AZM-7d group, while a trace amount of CARDS TX expression was still observable in the YQQFG-7d group.

## 4 Discussion

MPP is a prevalent form of childhood CAP and frequently causes outbreaks among student populations, thereby posing a significant public health challenge worldwide (Song et al., 2023). Currently, the treatment of MPP in children primarily involves symptomatic care and the use of macrolide antibiotics (Tsai et al., 2021). Nevertheless, in recent years, the resistance of MP to macrolide antibiotics has been increasing. This is especially true in East Asian nations like Japan and China, where the resistance rate in children is above 70% (Tanaka et al., 2017; Hsiung et al., 2022). This resistance often delays treatment, increasing the risk of progression from common to severe MPP (Lee et al., 2021). Clinically, tetracyclines and fluoroquinolones are considered alternative treatments (Tong et al., 2022). Unfortunately, the hazards of enamel hypoplasia and permanent tooth discolouration make tetracyclines unsafe for children under the age of eight. Patients under the age of 18 should exercise caution when prescribed fluoroquinolones due to the risk of musculoskeletal toxicity (Okubo et al., 2018). These challenges underline the urgent need for developing new therapeutic agents for MPP in children.

Pyroptosis, a form of programmed cell death, is characterized by the rupture of the cell membrane and the subsequent release of substantial inflammatory mediators (Liu et al., 2021). It is governed by both classical pathway (Caspase-1-dependent) and non-classical pathway (non-Caspase-1-dependent) mechanisms. (1) The classical pathway involves inflammasome activation of Caspase-1 (Dai et al., 2023). Inflammasomes, multiprotein complexes, consist of pattern recognition receptors (PRRs), an adaptor protein (ASC), and the precursor molecule (proCaspase-1) (Li et al., 2021). The human innate immune system mainly encompasses six types of PRRs, among which NOD-like receptors (NLRs) and absent in melanoma 2 (AIM2)-like receptors (ALRs) possess the capability to assemble inflammasomes (Man and Kanneganti, 2015; Isazadeh et al., 2023). Upon recognizing pathogen-associated molecular patterns (PAMPs) and damage-associated molecular patterns (DAMPs), NLRs or ALRs recruit ASC and proCaspase-1 via homotypic domain interactions to assemble the canonical inflammasome complexes, thereby converting proCaspase-1 into active Caspase-1 (Dick et al., 2016; Malik and Kanneganti, 2017). Caspase-1 not only converts the precursors of IL-1β and IL-18 into their mature forms but also cleaves GSDMD, producing the GSDMD-NT fragment that inserts into the lipid bilayer of the cell membrane, resulting in pyroptosis (Shi et al., 2015). (2) In the non-classical pathway, the bacterial endotoxin component lipopolysaccharide (LPS) commonly serves as the direct activator of Caspase-4, Caspase-5, or Caspase-11, which then cleave GSDMD to mediate pyroptosis (Kayagaki et al., 2013; Dai et al., 2023). Notably, this pathway does not directly augment the synthesis and release of IL-1β and IL-18, but rather promotes the release of ATP via the pannexin-1/P2X7 axis. Subsequently, ATP activates the NLRP3 inflammasome, thereby indirectly amplifying the inflammatory response (Yang et al., 2015; Li et al., 2023).

Among the various inflammasomes identified, the NLRP3 inflammasome attracts the most attention (Zhao and Zhao, 2020). A number of studies have shown that the classical pyroptosis pathway mediated by NLRP3 inflammasome plays an important role in acute lung injury induced by respiratory pathogens such as influenza and SARS-CoV-2 (Pan et al., 2021; Liu et al., 2022; You et al., 2023). The NLRP3 protein is a PRR found in the cytoplasm and is made up of three domains: an N-terminal pyrin domain, a central NACHT domain, and a C-terminal LRR domain (Seoane et al., 2020). At rest, NLRP3 is inactivated through binding with HSP90 and SGT1 in the cytoplasm (Yin et al., 2023). Upon pathogen invasion, the LRR domain senses danger signals, prompting NLRP3 to dissociate from HSP90 and SGT1, undergo oligomerization, expose its pyrin domain, and start recruiting ASC to assemble the inflammasome (Jo et al., 2016). IL-1β and IL-18, both characterized by their potent pro-inflammatory proFperties, are downstream products of the NLRP3 inflammasome and members of the IL-1 family. Due to the absence of signal peptides and leader sequences, IL-1β and IL-18 cannot be directly released into the extracellular environment via the Golgi vesicle transport pathway. Instead, their release necessitates cellular death mechanisms, such as pyroptosis (Lopez-Castejon and Brough, 2011). IL-1β is an important cytokine that mediates inflammatory damage in lung tissue. It can bind to the IL-1 receptor (IL-1R) on the surface of immune cells to promote the transcriptional expression of NF-κB, resulting in increased synthesis of pro-IL-1β, forming a positive feedback loop that escalates the inflammatory response (Vora et al., 2021). When IL-1β binds to IL-1R on lung vascular endothelial cells, it suppresses the transcription of VE-cadherin, compromising vascular integrity and causing lung vascular damage (Xiong et al., 2020). IL-18, initially identified as an interferon-γ (IFN-γ) inducing factor, targets T cells to induce IFN-γ production, promoting a Th1-type immune response (Yang et al., 2004). Furthermore, Narita M and colleagues have identified elevated levels of IL-18 in patients with MPP accompanied by pleural effusion or central nervous system complications, emphasizing the association between this cytokine and the severity of MPP disease (Narita et al., 2000). Hence, inhibiting NLRP3 inflammasome-mediated pyroptosis and reducing excessive levels of IL-1β and IL-18 constitute effective strategies for preventing and treating acute lung injury caused by MP.

Current studies have identified the pathogenesis of MP to primarily encompass direct adhesion damage, exotoxin release, immune inflammatory damage, and immune escape (Jiang et al., 2021). The most critical pathogenic mechanism is believed to be the intense immune inflammatory response triggered by the interaction between MP and the host (He et al., 2016). Given that macrophages are critical participants in the body’s inflammatory response (Knoll et al., 2021), this study confirms the therapeutic pathways and targets of YQQFG in treating MPP from the perspective of macrophage pyroptosis mediated by NLRP3 inflammasome, offering new insights into the prevention and treatment of MPP in children.

Initially, LC-MS/MS was utilized to identify compounds in YQQFG, from which 25 compounds with favorable oral bioavailability were selected. Notably, compounds such as Baicalein (Zhang et al., 2019; Zhang et al., 2020; Song et al., 2021), Quercetin (Colunga Biancatelli et al., 2020; Zheng et al., 2021; Sun et al., 2023), Eucalyptol (Zhao et al., 2014; Kennedy-Feitosa et al., 2016), Eugenol (Bittencourt-Mernak et al., 2021; Elken et al., 2022), Isorhamnetin (Zou et al., 2023), Luteolin (Kuo et al., 2011; Zhang et al., 2021), Gallic acid (Chosa et al., 1992; Haute et al., 2020), Naringenin (Lin et al., 2018; Zhang et al., 2022), 4-Hydroxycinnamic acid (Chen et al., 2019; Souza et al., 2021), and Hesperetin (Kaya et al., 2019; Wang et al., 2019) are recognized for their strong anti-inflammatory, antioxidant, and antimicrobial properties. The protective effects of these components against infectious lung injuries are well-documented. In MP-related studies, Chosa H et al. demonstrated significant bactericidal activity of Gallic Acid against MP (Chosa et al., 1992), while Lin Y et al. established that Naringenin prevents MP-induced pulmonary fibrosis by inhibiting autophagy in airway epithelial cells and reducing the inflammatory response in MPP model mice (Lin et al., 2018). These pharmacological findings provide support for verifying the mechanism of YQQFG in treating MPP.

Next, we utilized RAW264.7 cells and BALB/c mice to develop MP-infected cell and animal models, respectively. *In vitro*, peak production of IL-1β and TNF-α in RAW264.7 cells occurred 12 h post-MP infection, after which expression levels decreased. We speculated that 12 h post-MP intervention represents the peak of the pyroptotic effect, after which a significant number of cells have died. To test this hypothesis, we examined changes in cell morphology post-MP infection using SEM. MP is 1–2 μm in length and 0.1–0.2 μm in width, making it the smallest self-replicating organism currently identified, invisible to ordinary optical microscopes (Atkinson et al., 2008; Razin and Hayflick, 2010). We successfully captured the microscopic morphology of MP using SEM (Figure 3B). MP possesses a slender shape with a sharp structure at the top, which serves as its attachment organelle. This region harbors adhesion proteins and genomes that facilitate gliding, enabling MP to adhere to and colonize the cell surface (Krause, 1998; Balish, 2006). 12 h post-MP infection, we observed an increase in cell volume, cell membrane rupture, and the formation of numerous surface bubbles (Figures 3C, D), which are characteristic morphological features of pyroptosis (Xu et al., 2021). During the initial stage of pyroptosis, GSDMD inserts into the cell membrane, creating small pores. The osmotic pressure differential between the intracellular and extracellular environments allows only sodium ions and water from the extracellular matrix to enter, leading to cell enlargement and the appearance of bulging bubbles at the pores. As the cells continue to expand, the complete rupture of the cell membrane occurs, resulting in the release of cell contents and cell death (Dai et al., 2023). Subsequent WB and ELISA results indicated that in the Model group, the expression levels of NLRP3/Caspase-1/GSDMD pathway proteins and the concentrations of IL-1β and IL-18 were significantly increased, demonstrating that MP activates the NLRP3 inflammasome, triggering macrophage pyroptosis and the release of pro-inflammatory cytokines. Treatment with YQQFG-containing serum and AZM-containing serum reversed these changes, with no significant differences in the inhibition of the NLRP3/Caspase-1/GSDMD pathway. However, following NLRP3 overexpression, the lentivirus expressing NLRP3 neutralized the effect of AZM, whereas YQQFG-containing serum continued to suppress the levels of pyroptosis and the release of pro-inflammatory factors. This indicates that YQQFG’s therapeutic action is mediated through the regulation of NLRP3. It is well established that macrolide antibiotics function by binding to the 23S rRNA of MP ribosomes, thereby disrupting MP protein synthesis (Vázquez-Laslop and Mankin, 2018). However, mutations at specific sites on the 23S rRNA can prevent macrolides from binding effectively, leading to therapeutic failure (Leng et al., 2023). Different from the single-site mechanism of antibiotics against pathogens, TCM focuses on the overall regulation of the human body, so it is not easy to develop pathogen resistance, which fully reflects the significant potential of TCM in the prevention and treatment of infectious diseases.

Similar results were obtained *in vivo*. The lung index in MPP model mice increased, and H&E staining revealed acute inflammatory changes in lung tissue, such as extensive inflammatory cell infiltration, alveolar loss, and airway obstruction. WB and ELISA analyses indicated a significant increase in the expression levels of NLRP3/Caspase-1/GSDMD pathway proteins in the Model group, along with elevated levels of IL-1β and IL-18 in peripheral serum, and a strong co-localization of NLRP3 and F4/80. These results suggest that MP primarily activates the NLRP3 protein in macrophages, and this activation triggers macrophage pyroptosis, a key pathological mechanism in MP-induced acute lung injury. The expression of NLRP3 protein in macrophages significantly decreased after 3 days of YQQFG treatment. Following 7 days of treatment, there were marked improvements in lung index, inflammatory cell infiltration, and peripheral blood inflammation levels in mice treated with YQQFG. Thus, YQQFG appears to mitigate the inflammatory response by inhibiting NLRP3 inflammasome-mediated macrophage pyroptosis, effectively alleviating MP-induced acute lung injury. Importantly, our findings demonstrate that combining YQQFG with AZM yields the most effective therapeutic outcome for MPP, with synergistic effects observed. Moving forward, greater emphasis should be placed on integrating traditional Chinese and Western medicine in the treatment of MPP in children.

**FIGURE 11:**
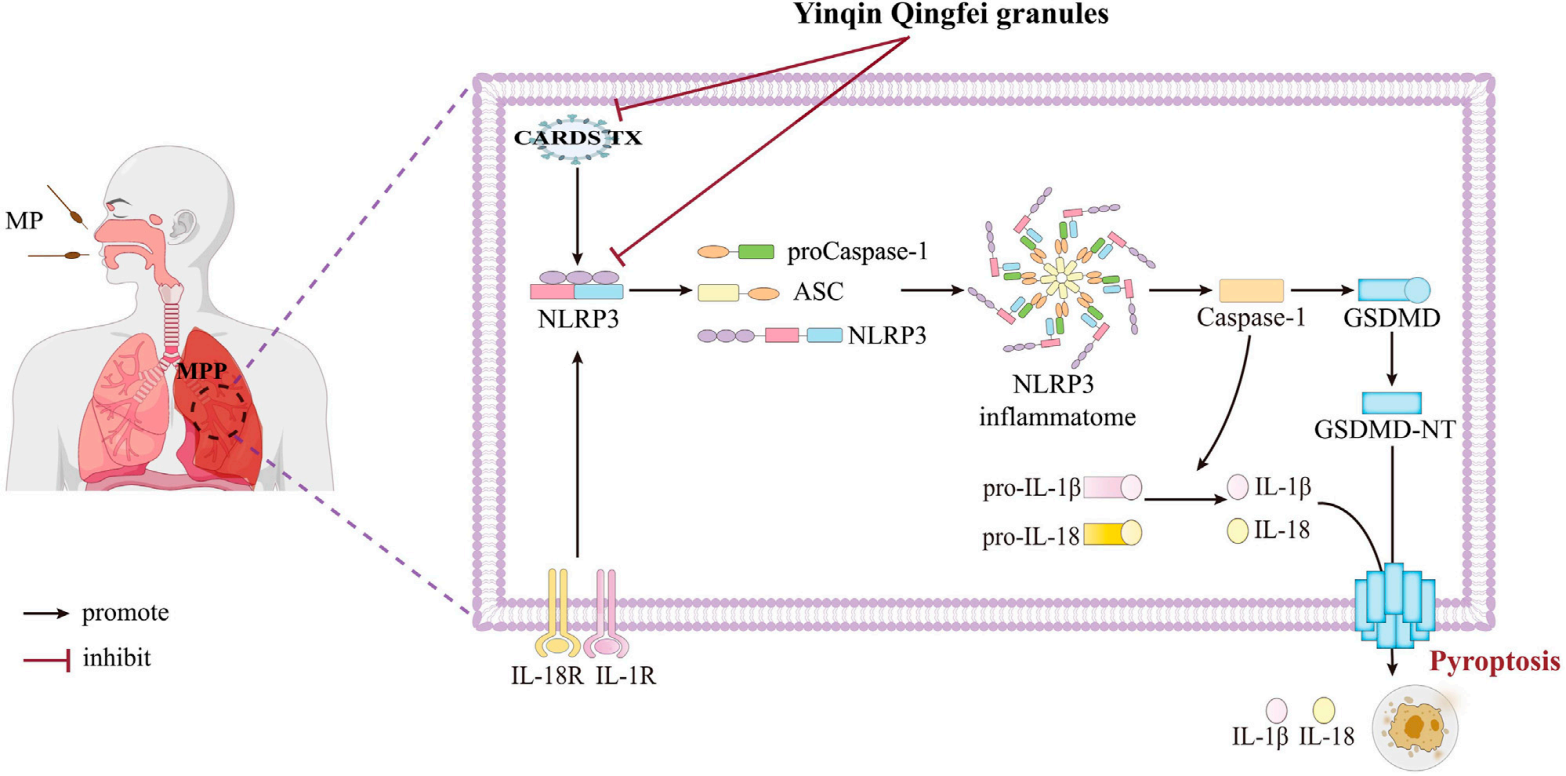
YQQFG treats MPP by inhibiting NLRP3 inflammasome-mediated macrophage pyroptosis. Some of the materials in this figure were sourced from Figdraw 2.0 (https://www.figdraw.com).

In 2005, Kannan TR et al. first identified the MP pathogenic factor MPN372, also known as CARDS TX (Kannan et al., 2005). Its structure closely resembles the S1 subunit of pertussis toxin and exhibits ADPRT activity and cell vacuolation activity (Hardy et al., 2009). CARDS TX is currently the only known exotoxin of MP and is strongly associated with MP’s pathogenicity and persistent infection (Techasaensiri et al., 2010). Given that CARDS TX can directly mediate the post-translational modification and activation of the NLRP3 protein (Bose et al., 2014), we assessed the relative expression of CARDS TX in various cell groups and mouse lung tissues using qPCR. The results indicated that YQQFG could reduce the release of CARDS TX to some extent, though not as effectively as AZM. This suggests that YQQFG not only targets the NLRP3 protein directly but also indirectly influences NLRP3 expression by inhibiting the effects of MP toxin (Figure 11).

Our research, however, has some limitations. First, while we have identified the main components of YQQFG, the key active components against MPP remain unclear. Second, the ability to treat diseases through a “multi-component, multi-target, and multi-pathway” approach is a distinctive advantage of TCM (Lou et al., 2022). Beyond NLRP3, other potential targets of YQQFG require further exploration and validation. Lastly, the interaction between YQQFG and AZM *in vivo* should be elucidated through pharmacokinetic studies to ensure the safety and efficacy of clinical use.

## 5 Conclusion

In summary, our study confirms that YQQFG mitigates the inflammatory response by inhibiting NLRP3 inflammasome-mediated macrophage pyroptosis, thereby alleviating MP-induced acute lung injury. This provides novel insights and approaches for the prevention and treatment of MPP in children.

## Figures and Tables

**Table:** 

**ADPRT**	ADP-ribosyltransferase
**AIM2**	absent in melanoma 2
**ALRs**	AIM2-like receptors
**AZM**	azithromycin
**BSL-2**	biosafety level-2
**CAP**	community-acquired pneumonia
**CARDS TX**	community-acquired respiratory distress syndrome toxin
**CCU**	color change units
**DAMPs**	damage-associated molecular patterns
**FBS**	fetal bovine serum
**GSDMD**	gasdermin D
**GSDMD-NT**	gasdermin D-N-terminal fragment
**IFN-γ**	interferon-γ
**IL-1β**	interleukin-1β
**IL-18**	interleukin-18
**IL-1R**	IL-1 receptor
**LV**	lentiviral
**MP**	Mycoplasma pneumoniae
**MPP**	Mycoplasma pneumoniae pneumonia
**NLRs**	NOD-like receptors
**OB**	oral bioavailability
**OD**	optical density
**PAMPs**	pathogen-associated molecular patterns
**PCC**	Pearson’s correlation coefficient
**PRRs**	pattern recognition receptors
**qPCR**	quantitative real-time PCR
**SEM**	scanning electron microscope
**TCM**	traditional Chinese medicine
**TIC**	total ion chromatogram
**TNF-α**	tumor necrosis factor-α
**TSA**	tyramide signal amplification
**WB**	western blot
**YQQFG**	Yinqin Qingfei granules

## Data Availability

The raw data presented in the study are deposited in the Figshare repository (https://doi.org/10.6084/m9.figshare.26784610.v2).

## References

[B1] Atkinson T. P. Balish M. F. Waites K. B. (2008). Epidemiology, clinical manifestations, pathogenesis and laboratory detection of Mycoplasma pneumoniae infections. FEMS Microbiol. Rev. 32 (6), 956–973. 10.1111/j.1574-6976.2008.00129.x 18754792

[B2] Balish M. F. (2006). Subcellular structures of mycoplasmas. Front. Biosci. 11, 2017–2027. 10.2741/1943 16720287

[B3] Bittencourt-Mernak M. I. Pinheiro N. M. da Silva R. C. Ponci V. Banzato R. Pinheiro A. (2021). Effects of Eugenol and dehydrodieugenol B from nectandra leucantha against lipopolysaccharide (LPS)-Induced experimental acute lung inflammation. J. Nat. Prod. 84 (8), 2282–2294. 10.1021/acs.jnatprod.1c00386 34264084

[B4] Bose S. Segovia J. A. Somarajan S. R. Chang T. H. Kannan T. R. Baseman J. B. (2014). ADP-ribosylation of NLRP3 by Mycoplasma pneumoniae CARDS toxin regulates inflammasome activity. mBio 5 (6). 10.1128/mBio.02186-14 PMC4278538 25538194

[B5] Byrne A. J. Mathie S. A. Gregory L. G. Lloyd C. M. (2015). Pulmonary macrophages: key players in the innate defence of the airways. Thorax 70 (12), 1189–1196. 10.1136/thoraxjnl-2015-207020 26286722

[B6] Calus D. Maes D. Vranckx K. Villareal I. Pasmans F. Haesebrouck F. (2010). Validation of ATP luminometry for rapid and accurate titration of Mycoplasma hyopneumoniae in Friis medium and a comparison with the color changing units assay. J. Microbiol. Methods 83 (3), 335–340. 10.1016/j.mimet.2010.09.001 20851152

[B7] Chen J. J. Deng J. S. Huang C. C. Li P. Y. Liang Y. C. Chou C. Y. (2019). p-Coumaric-Acid-Containing adenostemma lavenia ameliorates acute lung injury by activating AMPK/Nrf2/HO-1 signaling and improving the anti-oxidant response. Am. J. Chin. Med. 47 (7), 1483–1506. 10.1142/s0192415x19500769 31645126

[B8] Chosa H. Toda M. Okubo S. Hara Y. Shimamura T. (1992). Antimicrobial and microbicidal activities of tea and catechins against Mycoplasma. Kansenshogaku Zasshi 66 (5), 606–611. 10.11150/kansenshogakuzasshi1970.66.606 1402093

[B9] Colunga Biancatelli R. M. L. Berrill M. Catravas J. D. Marik P. E. (2020). Quercetin and vitamin C: an experimental, synergistic therapy for the prevention and treatment of SARS-CoV-2 related disease (COVID-19). Front. Immunol. 11, 1451. 10.3389/fimmu.2020.01451 32636851 PMC7318306

[B10] Dai Z. Liu W. C. Chen X. Y. Wang X. Li J. L. Zhang X. (2023). Gasdermin D-mediated pyroptosis: mechanisms, diseases, and inhibitors. Front. Immunol. 14, 1178662. 10.3389/fimmu.2023.1178662 37275856 PMC10232970

[B11] Deng F. Cao H. L. Liang X. H. Li Q. B. Yang Y. Zhao Z. H. (2023). Analysis of cytokine levels, cytological findings, and MP-DNA level in bronchoalveolar lavage fluid of children with Mycoplasma pneumoniae pneumonia. Immun. Inflamm. Dis. 11 (5), e849. 10.1002/iid3.849 37249293 PMC10165957

[B79] Dick M. S. Sborgi L. Rühl S. Hiller S. Broz P. (2016). ASC filament formation serves as a signal amplification mechanism for inflammasomes. Nat. Commun. 7, 11929. 10.1038/ncomms11929 27329339 PMC4917984

[B12] Ding X. Kambara H. Guo R. Kanneganti A. Acosta-Zaldívar M. Li J. (2021). Inflammasome-mediated GSDMD activation facilitates escape of Candida albicans from macrophages. Nat. Commun. 12 (1), 6699. 10.1038/s41467-021-27034-9 34795266 PMC8602704

[B13] Dou Y. Q. Ma T. Li Z. J. Ma X. R. Zhang C. J. Guo Q. N. (2024). Buyang Huanwu Decoction promotes arteriogenesis after hindlimb ischemia in mice by activating PDGF signaling pathway. Zhongguo Zhong Yao Za Zhi 49 (1), 216–223. 10.19540/j.cnki.cjcmm.20230919.402 38403354

[B14] Dunn K. W. Kamocka M. M. McDonald J. H. (2011). A practical guide to evaluating colocalization in biological microscopy. Am. J. Physiol. Cell Physiol. 300 (4), C723–C742. 10.1152/ajpcell.00462.2010 21209361 PMC3074624

[B15] Elken E. M. Tan Z. N. Wang Q. Jiang X. Y. Wang Y. Wang Y. M. (2022). Impact of sub-MIC Eugenol on *Klebsiella pneumoniae* biofilm formation via upregulation of rcsB. Front. Vet. Sci. 9, 945491. 10.3389/fvets.2022.945491 35903134 PMC9315372

[B16] Fan E. K. Y. Fan J. (2018). Regulation of alveolar macrophage death in acute lung inflammation. Respir. Res. 19 (1), 50. 10.1186/s12931-018-0756-5 29587748 PMC5872399

[B17] Fu J. Wu H. (2023). Structural mechanisms of NLRP3 inflammasome assembly and activation. Annu. Rev. Immunol. 41, 301–316. 10.1146/annurev-immunol-081022-021207 36750315 PMC10159982

[B18] Hardy R. D. Coalson J. J. Peters J. Chaparro A. Techasaensiri C. Cantwell A. M. (2009). Analysis of pulmonary inflammation and function in the mouse and baboon after exposure to Mycoplasma pneumoniae CARDS toxin. PLoS One 4 (10), e7562. 10.1371/journal.pone.0007562 19859545 PMC2762541

[B19] Haute G. V. Luft C. Antunes G. L. Silveira J. S. de Souza Basso B. da Costa M. S. (2020). Anti-inflammatory effect of octyl gallate in alveolar macrophages cells and mice with acute lung injury. J. Cell Physiol. 235 (9), 6073–6084. 10.1002/jcp.29536 31970778

[B20] He J. Liu M. Ye Z. Tan T. Liu X. You X. (2016). Insights into the pathogenesis of mycoplasma pneumoniae (review). Mol. Med. Rep. 14 (5), 4030–4036. 10.3892/mmr.2016.5765 27667580 PMC5101875

[B21] Hsiung J. C. C. Ma H. Y. Lu C. Y. Yen T. Y. Chi H. Liau Y. J. (2022). Children with Mycoplasma pneumoniae infection in Taiwan: changes in molecular characteristics and clinical outcomes. J. Formos. Med. Assoc. 121 (11), 2273–2280. 10.1016/j.jfma.2022.05.001 35599105

[B76] Huo J. Wang T. Wei B. Shi X. Yang A. Chen D. (2022). Integrated network pharmacology and intestinal flora analysis to determine the protective effect of Xuanbai-Chengqi decoction on lung and gut injuries in influenza virus-infected mice. J. Ethnopharmacol. 298, 115649. 10.1016/j.jep.2022.115649 35987410

[B22] Isazadeh A. Heris J. A. Shahabi P. Mohammadinasab R. Shomali N. Nasiri H. (2023). Pattern-recognition receptors (PRRs) in SARS-CoV-2. Life Sci. 329, 121940. 10.1016/j.lfs.2023.121940 37451397

[B23] Jacobs E. Ehrhardt I. Dumke R. (2015). New insights in the outbreak pattern of Mycoplasma pneumoniae. Int. J. Med. Microbiol. 305 (7), 705–708. 10.1016/j.ijmm.2015.08.021 26319941

[B24] Ji L. Song T. Ge C. Wu Q. Ma L. Chen X. (2023). Identification of bioactive compounds and potential mechanisms of scutellariae radix-coptidis rhizoma in the treatment of atherosclerosis by integrating network pharmacology and experimental validation. Biomed. Pharmacother. 165, 115210. 10.1016/j.biopha.2023.115210 37499457

[B25] Jiang Z. L. Li S. H. Zhu C. M. Zhou R. J. Leung P. H. M. (2021). Mycoplasma pneumoniae infections: pathogenesis and vaccine development. Pathogens 10 (2), 119. 10.3390/pathogens10020119 33503845 PMC7911756

[B85] Jo E. K. Kim J. K. Shin D. M. Sasakawa C. (2016). Molecular mechanisms regulating NLRP3 inflammasome activation. Cell Mol. Immunol. 13 (148), 159. 10.1038/cmi.2015.95 PMC4786634 26549800

[B26] Kannan T. R. Provenzano D. Wright J. R. Baseman J. B. (2005). Identification and characterization of human surfactant protein A binding protein of Mycoplasma pneumoniae. Infect. Immun. 73 (5), 2828–2834. 10.1128/iai.73.5.2828-2834.2005 15845487 PMC1087375

[B27] Kaya S. Albayrak Kaya S. Polat E. Fidanol Erboğa Z. Duran Y. Polat F. R. (2019). Protective effects of hesperetin on lipopolysaccharide-induced acute lung injury in a rat model. Turk Gogus Kalp Damar Cerrahisi Derg. 28 (2), 359–368. 10.5606/tgkdc.dergisi.2020.18816 32551168 PMC7298383

[B81] Kayagaki N. Wong M. T. Stowe I. B. Ramani S. R. Gonzalez L. C. Akashi-Takamura S. (2013). Noncanonical inflammasome activation by intracellular LPS independent of TLR4. Science 341 (6151), 1246–1249. 10.1126/science.1240248 23887873

[B28] Kennedy-Feitosa E. Okuro R. T. Pinho Ribeiro V. Lanzetti M. Barroso M. V. Zin W. A. (2016). Eucalyptol attenuates cigarette smoke-induced acute lung inflammation and oxidative stress in the mouse. Pulm. Pharmacol. Ther. 41, 11–18. 10.1016/j.pupt.2016.09.004 27599597

[B29] Kim K. Jung S. Kim M. Park S. Yang H. J. Lee E. (2022). Global trends in the proportion of macrolide-resistant mycoplasma pneumoniae infections: a systematic review and meta-analysis. JAMA Netw. Open 5 (7), e2220949. 10.1001/jamanetworkopen.2022.20949 35816304 PMC9274321

[B30] Knoll R. Schultze J. L. Schulte-Schrepping J. (2021). Monocytes and macrophages in COVID-19. Front. Immunol. 12, 720109. 10.3389/fimmu.2021.720109 34367190 PMC8335157

[B31] Krause D. C. (1998). Mycoplasma pneumoniae cytadherence: organization and assembly of the attachment organelle. Trends Microbiol. 6 (1), 15–18. 10.1016/s0966-842x(97)01168-2 9481818

[B32] Kumar S. (2018). Mycoplasma pneumoniae: a significant but underrated pathogen in paediatric community-acquired lower respiratory tract infections. Indian J. Med. Res. 147, 23–31. 10.4103/ijmr.IJMR_1582_16 29749357 PMC5967212

[B33] Kumar S. Kumar S. (2023). Mycoplasma pneumoniae: among the smallest bacterial pathogens with great clinical significance in children. Indian J. Med. Microbiol. 46, 100480. 10.1016/j.ijmmb.2023.100480 37741157

[B34] Kuo M. Y. Liao M. F. Chen F. L. Li Y. C. Yang M. L. Lin R. H. (2011). Luteolin attenuates the pulmonary inflammatory response involves abilities of antioxidation and inhibition of MAPK and NFκB pathways in mice with endotoxin-induced acute lung injury. Food Chem. Toxicol. 49 (10), 2660–2666. 10.1016/j.fct.2011.07.012 21782879

[B35] Lee K. L. Lee C. M. Yang T. L. Yen T. Y. Chang L. Y. Chen J. M. (2021). Severe Mycoplasma pneumoniae pneumonia requiring intensive care in children, 2010-2019. J. Formos. Med. Assoc. 120 (1 Pt 1), 281–291. 10.1016/j.jfma.2020.08.018 32948415

[B36] Leng M. Yang J. Zhou J. (2023). The molecular characteristics, diagnosis, and treatment of macrolide-resistant Mycoplasma pneumoniae in children. Front. Pediatr. 11, 1115009. 10.3389/fped.2023.1115009 36937963 PMC10017863

[B37] Li D. M. Tang S. H. Liao Q. Chen W. Zhang H. C. (2017). Literature study on prevention and treatment of community acquired pneumonia by traditional Chinese medicine. Zhongguo Zhong Yao Za Zhi 42 (8), 1418–1422. 10.19540/j.cnki.cjcmm.2017.0037 29071842

[B38] Li G. Fan L. Wang Y. Huang L. Wang M. Zhu C. (2019). High co-expression of TNF-α and CARDS toxin is a good predictor for refractory Mycoplasma pneumoniae pneumonia. Mol. Med. 25 (1), 38. 10.1186/s10020-019-0105-2 31399022 PMC6688229

[B78] Li Y. Huang H. Liu B. Zhang Y. Pan X. Yu X. Y. (2021). Inflammasomes as therapeutic targets in human diseases. Signal Transduct. Target Ther 6 (1), 247. 10.1038/s41392-021-00650-z 34210954 PMC8249422

[B83] Li Z. Li D. Chen R. Gao S. Xu Z. Li N. (2023). Cell death regulation: a new way for natural products to treat osteoporosis. Pharmacol. Res. 187, 106635. 10.1016/j.phrs.2022.106635 36581167

[B39] Lin Y. Tan D. Kan Q. Xiao Z. Jiang Z. (2018). The protective effect of Naringenin on airway remodeling after mycoplasma pneumoniae infection by inhibiting autophagy-mediated lung inflammation and fibrosis. Mediat. Inflamm. 2018, 8753894. 10.1155/2018/8753894 PMC5904783 29849498

[B77] Liu X. Xia S. Zhang Z. Wu H. Lieberman J. (2021). Channelling inflammation: gasdermins in physiology and disease. Nat. Rev. Drug. Discov. 20 (5), 384–405. 10.1038/s41573-021-00154-z 33692549 PMC7944254

[B40] Liu X. Lin Z. Yin X. (2022). Pellino2 accelerate inflammation and pyroptosis via the ubiquitination and activation of NLRP3 inflammation in model of pediatric pneumonia. Int. Immunopharmacol. 110, 108993. 10.1016/j.intimp.2022.108993 35809381

[B87] Lopez-Castejon G. Brough D. (2011). Understanding the mechanism of IL-1β secretion. Cytokine Growth Factor Rev. 22 (4), 189–195. 10.1016/j.cytogfr.2011.10.001 22019906 PMC3714593

[B41] Lou Y. Ma M. Jiang Y. Xu H. Gao Z. Gao L. (2022). Ferroptosis: a new strategy for traditional Chinese medicine treatment of stroke. Biomed. Pharmacother. 156, 113806. 10.1016/j.biopha.2022.113806 36228377

[B42] Luo H. He J. Qin L. Chen Y. Chen L. Li R. (2021). Mycoplasma pneumoniae lipids license TLR-4 for activation of NLRP3 inflammasome and autophagy to evoke a proinflammatory response. Clin. Exp. Immunol. 203 (1), 66–79. 10.1111/cei.13510 32894580 PMC7744503

[B43] Man S. M. Kanneganti T. D. (2015). Regulation of inflammasome activation. Immunol. Rev. 265 (1), 6–21. 10.1111/imr.12296 25879280 PMC4400844

[B80] Malik A. Kanneganti T. D. (2017). Inflammasome activation and assembly at a glance. J. Cell Sci. 130 (23), 3955–3963. 10.1242/jcs.207365 29196474 PMC5769591

[B44] Narita M. (2010). Pathogenesis of extrapulmonary manifestations of Mycoplasma pneumoniae infection with special reference to pneumonia. J. Infect. Chemother. 16 (3), 162–169. 10.1007/s10156-010-0044-x 20186455

[B90] Narita M. Tanaka H. Abe S. Yamada S. Kubota M. Togashi T. (2000). Close association between pulmonary disease manifestation in Mycoplasma pneumoniae infection and enhanced local production of interleukin-18 in the lung, independent of gamma interferon. Clin. Diagn. Lab. Immunol. 7 (6), 909–914. 10.1128/CDLI.7.6.909-914.2000 11063497 PMC95984

[B45] Okubo Y. Michihata N. Morisaki N. Uda K. Miyairi I. Ogawa Y. (2018). Recent trends in practice patterns and impact of corticosteroid use on pediatric Mycoplasma pneumoniae-related respiratory infections. Respir. Investig. 56 (2), 158–165. 10.1016/j.resinv.2017.11.005 29548654

[B46] Pan P. Shen M. Yu Z. Ge W. Chen K. Tian M. (2021). SARS-CoV-2 N protein promotes NLRP3 inflammasome activation to induce hyperinflammation. Nat. Commun. 12 (1), 4664. 10.1038/s41467-021-25015-6 34341353 PMC8329225

[B47] Razin S. Hayflick L. (2010). Highlights of mycoplasma research--an historical perspective. Biologicals 38 (2), 183–190. 10.1016/j.biologicals.2009.11.008 20149687

[B48] Seoane P. I. Lee B. Hoyle C. Yu S. Lopez-Castejon G. Lowe M. (2020). The NLRP3-inflammasome as a sensor of organelle dysfunction. J. Cell Biol. 219 (12), e202006194. 10.1083/jcb.202006194 33044555 PMC7543090

[B49] Shi J. Zhao Y. Wang K. Shi X. Wang Y. Huang H. (2015). Cleavage of GSDMD by inflammatory caspases determines pyroptotic cell death. Nature 526 (7575), 660–665. 10.1038/nature15514 26375003

[B50] Song J. Zhang L. Xu Y. Yang D. Zhang L. Yang S. (2021). The comprehensive study on the therapeutic effects of baicalein for the treatment of COVID-19 *in vivo* and *in vitro* . Biochem. Pharmacol. 183, 114302. 10.1016/j.bcp.2020.114302 33121927 PMC7588320

[B51] Song Z. Jia G. Luo G. Han C. Zhang B. Wang X. (2023). Global research trends of Mycoplasma pneumoniae pneumonia in children: a bibliometric analysis. Front. Pediatr. 11, 1306234. 10.3389/fped.2023.1306234 38078315 PMC10704248

[B52] Souza T. N. Santos F. M. Alves P. R. Ferro J. N. Correia A. C. C. Melo T. S. (2021). Local administration of p-coumaric acid decreases lipopolysaccharide-induced acute lung injury in mice: *in vitro* and *in silico* studies. Eur. J. Pharmacol. 897, 173929. 10.1016/j.ejphar.2021.173929 33561444

[B53] Sun J. H. Sun F. Yan B. Li J. Y. Xin L. (2020). Data mining and systematic pharmacology to reveal the mechanisms of traditional Chinese medicine in Mycoplasma pneumoniae pneumonia treatment. Biomed. Pharmacother. 125, 109900. 10.1016/j.biopha.2020.109900 32028237

[B54] Sun Y. L. Zhao P. P. Zhu C. B. Jiang M. C. Li X. M. Tao J. L. (2023). Integrating metabolomics and network pharmacology to assess the effects of quercetin on lung inflammatory injury induced by human respiratory syncytial virus. Sci. Rep. 13 (1), 8051. 10.1038/s41598-023-35272-8 37198253 PMC10192330

[B55] Tanaka T. Oishi T. Miyata I. Wakabayashi S. Kono M. Ono S. (2017). Macrolide-Resistant mycoplasma pneumoniae infection, Japan, 2008-2015. Emerg. Infect. Dis. 23 (10), 1703–1706. 10.3201/eid2310.170106 28930026 PMC5621555

[B56] Techasaensiri C. Tagliabue C. Cagle M. Iranpour P. Katz K. Kannan T. R. (2010). Variation in colonization, ADP-ribosylating and vacuolating cytotoxin, and pulmonary disease severity among mycoplasma pneumoniae strains. Am. J. Respir. Crit. Care Med. 182 (6), 797–804. 10.1164/rccm.201001-0080OC 20508214 PMC2949405

[B57] Tong L. Huang S. Zheng C. Zhang Y. Chen Z. (2022). Refractory mycoplasma pneumoniae pneumonia in children: early recognition and management. J. Clin. Med. 11 (10), 2824. 10.3390/jcm11102824 35628949 PMC9144103

[B58] Tsai T. A. Tsai C. K. Kuo K. C. Yu H. R. (2021). Rational stepwise approach for Mycoplasma pneumoniae pneumonia in children. J. Microbiol. Immunol. Infect. 54 (4), 557–565. 10.1016/j.jmii.2020.10.002 33268306

[B59] Vázquez-Laslop N. Mankin A. S. (2018). How macrolide antibiotics work. Trends Biochem. Sci. 43 (9), 668–684. 10.1016/j.tibs.2018.06.011 30054232 PMC6108949

[B88] Vora S. M. Lieberman J. Wu H. (2021). Inflammasome activation at the crux of severe COVID-19. Nat. Rev. Immunol. 21 (11), 694–703. 10.1038/s41577-021-00588-x 34373622 PMC8351223

[B60] Wang N. Geng C. Sun H. Wang X. Li F. Liu X. (2019). Hesperetin ameliorates lipopolysaccharide-induced acute lung injury in mice through regulating the TLR4-MyD88-NF-κB signaling pathway. Arch. Pharm. Res. 42 (12), 1063–1070. 10.1007/s12272-019-01200-6 31802426

[B61] Wang Y. Ye Q. Yang D. Ni Z. Chen Z. (2016). Study of two separate types of macrolide-resistant mycoplasma pneumoniae outbreaks. Antimicrob. Agents Chemother. 60 (7), 4310–4314. 10.1128/aac.00198-16 27161643 PMC4914620

[B89] Xiong S. Hong Z. Huang L. S. Tsukasaki Y. Nepal S. Di A. (2020). IL-1β suppression of VE-cadherin transcription underlies sepsis-induced inflammatory lung injury. J. Clin. Invest. 130 (7), 3684–3698. 10.1172/JCI136908 32298238 PMC7324198

[B62] Xu L. Wang H. Yu Q. Q. Ge J. R. Zhang X. Z. Mei D. (2021). The monomer derivative of paeoniflorin inhibits macrophage pyroptosis via regulating TLR4/NLRP3/GSDMD signaling pathway in adjuvant arthritis rats. Int. Immunopharmacol. 101 (Pt A), 108169. 10.1016/j.intimp.2021.108169 34607227

[B63] Xu X. Zhang W. Huang C. Li Y. Yu H. Wang Y. (2012). A novel chemometric method for the prediction of human oral bioavailability. Int. J. Mol. Sci. 13 (6), 6964–6982. 10.3390/ijms13066964 22837674 PMC3397506

[B82] Yang D. He Y. Muñoz-Planillo R. Liu Q. Núñez G. (2015). Caspase-11 Requires the Pannexin-1 Channel and the Purinergic P2X7 Pore to Mediate Pyroptosis and Endotoxic Shock. Immunity 43 (5), 923–932. 10.1016/j.immuni.2015.10.009 26572062 PMC4795157

[B64] Yin M. Marrone L. Peace C. G. O'Neill L. A. J. (2023). NLRP3, the inflammasome and COVID-19 infection. Qjm 116 (7), 502–507. 10.1093/qjmed/hcad011 36661317 PMC10382191

[B86] Yang J. Hooper W. C. Phillips D. J. Talkington D. F. (2004). Cytokines in Mycoplasma pneumoniae infections. Cytokine Growth Factor Rev. 15 (2–3), 157–168. 10.1016/j.cytogfr.2004.01.001 15110799

[B65] You J. Zhou L. San X. Li H. Li M. Wang B. (2023). NEDD4 regulated pyroptosis occurred from Co-infection between influenza A virus and Streptococcus pneumoniae. J. Microbiol. 61 (8), 777–789. 10.1007/s12275-023-00076-y 37792248

[B75] Zheng C. Pei T. Huang C. Chen Y. Bai M. Xue J. (2016). A novel systems pharmacology platform to dissect action mechanisms of traditional Chinese medicines for bovine viral diarrhea disease. Eur. J. Pharm. Sci. 94, 33–45. 10.1016/j.ejps.2016.05.018 27208435

[B66] Zhang C. Li N. Niu F. (2019). Baicalein triazole prevents respiratory tract infection by RSV through suppression of oxidative damage. Microb. Pathog. 131, 227–233. 10.1016/j.micpath.2019.03.026 30943433

[B67] Zhang H. Luan Y. Jing S. Wang Y. Gao Z. Yang P. (2020). Baicalein mediates protection against Staphylococcus aureus-induced pneumonia by inhibiting the coagulase activity of vWbp. Biochem. Pharmacol. 178, 114024. 10.1016/j.bcp.2020.114024 32413427

[B68] Zhang X. Li M. Wu H. Fan W. Zhang J. Su W. (2022). Naringenin attenuates inflammation, apoptosis, and ferroptosis in silver nanoparticle-induced lung injury through a mechanism associated with Nrf2/HO-1 axis: *in vitro* and *in vivo* studies. Life Sci. 311 (Pt A), 121127. 10.1016/j.lfs.2022.121127 36306867

[B69] Zhang Z. T. Zhang D. Y. Xie K. Wang C. J. Xu F. (2021). Luteolin activates Tregs to promote IL-10 expression and alleviating caspase-11-dependent pyroptosis in sepsis-induced lung injury. Int. Immunopharmacol. 99, 107914. 10.1016/j.intimp.2021.107914 34246059

[B70] Zhao C. Sun J. Fang C. Tang F. (2014). 1,8-cineol attenuates LPS-induced acute pulmonary inflammation in mice. Inflammation 37 (2), 566–572. 10.1007/s10753-013-9770-4 24197825

[B84] Zhao C. Zhao W. (2020). NLRP3 inflammasome-a key player in antiviral responses. Front. Immunol. 11, 211. 10.3389/fimmu.2020.00211 32133002 PMC7040071

[B71] Zheng W. Wu H. Wang T. Zhan S. Liu X. (2021). Quercetin for COVID-19 and DENGUE co-infection: a potential therapeutic strategy of targeting critical host signal pathways triggered by SARS-CoV-2 and DENV. Brief. Bioinform 22 (6), bbab199. 10.1093/bib/bbab199 34058750 PMC8195157

[B72] Zou Y. Wang H. Fang J. Sun H. Deng X. Wang J. (2023). Isorhamnetin as a novel inhibitor of pneumolysin against Streptococcus pneumoniae infection *in vivo*/*in vitro* . Microb. Pathog. 185, 106382. 10.1016/j.micpath.2023.106382 37839759

