# Supplementary material for: Yinqin Qingfei granules alleviate Mycoplasma pneumoniae pneumonia via inhibiting NLRP3 inflammasome-mediated macrophage pyroptosis
Source: Front Pharmacol. 2024 Aug 27;15:1437475. doi: 10.3389/fphar.2024.1437475 (PMC11383775; doi:10.3389/fphar.2024.1437475)

**Determination of MP concentration by CCU**

The MP liquid was serially diluted and observed continuously for one month. The highest dilution unit that caused color change was 10^-8^. The concentration of MP we cultured was 5 × 10^8^ CCU/mL.


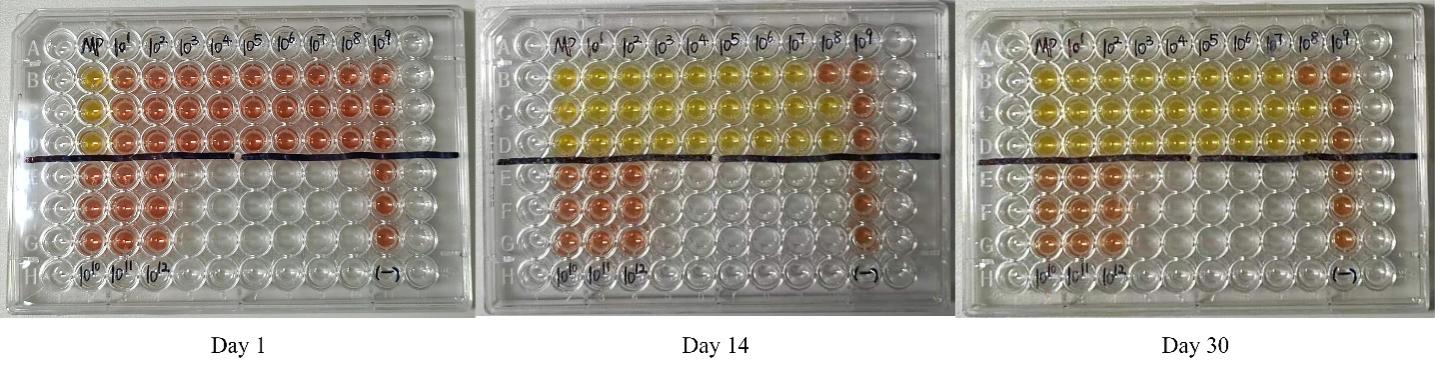

Supplement: Supplementary file 2 [file DataSheet2.DOCX]
