# Supplementary material for: Yinqin Qingfei granules alleviate Mycoplasma pneumoniae pneumonia via inhibiting NLRP3 inflammasome-mediated macrophage pyroptosis
Source: Front Pharmacol. 2024 Aug 27;15:1437475. doi: 10.3389/fphar.2024.1437475 (PMC11383775; doi:10.3389/fphar.2024.1437475)
Supplement: Supplementary file 3 [file DataSheet1.DOCX]

**Specific experimental parameters of LC-MS/MS**

Liquid chromatography conditions: The LC analysis was performed on a Vanquish UHPLC System (Thermo Fisher Scientific, USA). Chromatography was carried out with an ACQUITY UPLC ^®^ HSS T3 (2.1×100 mm, 1.8 µm) (Waters, Milford, MA, USA). The column maintained at 40℃. The flow rate and injection volume were set at 0.3 mL/min and 2 μL, respectively. For LC-ESI (+)-MS analysis, the mobile phases consisted of (B2) 0.1% formic acid in acetonitrile (v/v) and (A2) 0.1% formic acid in water (v/v). Separation was conducted under the following gradient: 0~1 min, 8% B2; 1~8 min, 8%~98% B2; 8~10 min, 98% B2; 10~10.1 min, 98%~8% B2; 10.1~12 min, 8% B2. For LC-ESI (-)-MS analysis, the analytes was carried out with (B3) acetonitrile and (A3) ammonium formate (5mM). Separation was conducted under the following gradient: 0~1 min, 8% B3; 1~8 min, 8%~98% B3; 8~10 min, 98% B3; 10~10.1 min, 98%~8% B3; 10.1~12 min, 8% B3.

Mass spectrum conditions: Mass spectrometric detection of metabolites was performed on Q Exactive (Thermo Fisher Scientific, USA) with ESI ion source. Simultaneous MS1 and MS/MS (Full MS-ddMS2 mode, data-dependent MS/MS) acquisition was used. The parameters were as follows: sheath gas pressure, 40 arb; aux gas flow, 10 arb; spray voltage, 3.50 kV and -2.50 kV for ESI(+) and ESI(-), respectively; capillary temperature, 325℃; MS1 range, m/z 100-1000; MS1 resolving power, 70000 FWHM; number of data dependant scans per cycle, 10; MS/MS resolving power, 17500 FWHM; normalized collision energy, 30 eV; dynamic exclusion time, automatic.
